# Studies on Biological and Molecular Effects of Small-Molecule Kinase Inhibitors on Human Glioblastoma Cells and Organotypic Brain Slices

**DOI:** 10.3390/life12081258

**Published:** 2022-08-17

**Authors:** Julia Hörnschemeyer, Timo Kirschstein, Gesine Reichart, Christin Sasse, Jakob Venus, Anne Einsle, Katrin Porath, Michael Linnebacher, Rüdiger Köhling, Falko Lange

**Affiliations:** 1 Oscar-Langendorff-Institute of Physiology, Rostock University Medical Center, 18057 Rostock, Germany; 2 Center for Transdisciplinary Neurosciences Rostock, University of Rostock, 18147 Rostock, Germany; 3 Clinic for General Surgery, Molecular Oncology and Immunotherapy, Rostock University Medical Center, 18057 Rostock, Germany

**Keywords:** glioblastoma, low-passage glioblastoma cell lines, ipatasertib, MK-2206, dactolisib, trametinib, regorafenib, organotypic brain slice cultures

## Abstract

Glioblastoma is the most common and aggressive primary brain tumor. Multiple genetic and epigenetic alterations in several major signaling pathways—including the phosphoinositide 3-kinases (PI3K)/AKT/mTOR and the Raf/MEK/ERK pathway—could be found. We therefore aimed to investigate the biological and molecular effects of small-molecule kinase inhibitors that may interfere with those pathways. For this purpose, patient-derived glioblastoma cells were challenged with dactolisib, ipatasertib, MK-2206, regorafenib, or trametinib. To determine the effects of the small-molecule kinase inhibitors, assays of cell proliferation and apoptosis and immunoblot analyses were performed. To further investigate the effects of ipatasertib on organotypic brain slices harboring glioblastoma cells, the tumor growth was estimated. In addition, the network activity in brain slices was assessed by electrophysiological field potential recordings. Multi-kinase inhibitor regorafenib and both MK-2206 and dactolisib were very effective in all preclinical tumor models, while with respect to trametinib, two cell lines were found to be highly resistant. Only in HROG05 cells, ipatasertib showed anti-tumoral effects in vitro and in organotypic brain slices. Additionally, ipatasertib diminished synchronous network activity in organotypic brain slices. Overall, our data suggest that ipatasertib was only effective in selected tumor models, while especially regorafenib and MK-2206 presented a uniform response pattern.

## 1. Introduction

Glioblastoma (WHO grade IV glioma) is the most common malignant tumor in the central nervous system and has one of the worst survival prognoses of all tumor diseases [1]. Most of the glioblastomas arise de novo as primary tumors, whereas a small number is secondary derived from less-malignant precursor lesions. Gliomagenesis is accompanied by a multitude of genetic and epigenetic alterations leading to different molecular subtypes of the disease [2,3].

Genes encoding for members of the phosphatidylinositol-3-kinase (PI3K)/AKT/ mammalian target of rapamycin (mTOR) pathway are often found to be affected by tumorigenic alterations [4,5]. While PI3K gain-of-function mutations were detected in 25% of patients suffering from glioblastoma, the main negative regulator of the PI3K/AKT/mTOR pathway, the phosphatase and tensin homolog (PTEN), is deleted or affected by loss-of-function mutations in 40% of the cases. In addition, mutations are also found in the mitogen-activated protein kinase (MAPK) pathway. Brennan et al. (2013) detected RAS mutations in 1% of the cases and BRAF mutations in 2% [4], while upstream receptor tyrosine kinases (RTK) such as the epidermal growth factor (EGF) receptor were overexpressed or mutated in more than half of the investigated tumor samples [4]. The high prevalence of genetic changes in both signaling pathways singles them out as potential therapeutic targets of small-molecule kinase inhibitors (SMI). Both single-kinase and multi-kinase inhibitors have attracted attention and were tested in the present study.

With respect to the PI3K/AKT/mTOR pathway, the dual PI3K/mTOR inhibitor dactolisib attenuated tumor progression in animal models of glioma [6,7], and the allosteric AKT inhibitor MK-2206 presented antitumoral effects in vitro [8,9]. Regarding the MAPK signaling pathway, inhibition of mitogen-activated protein kinase kinase (MEK) by trametinib contributed to mitigating effects on glioblastoma cell growth in preclinical models [10] and was used in combination with dabrafenib in a clinical trial treating glioblastoma with BRAF^V600E^ mutations [11]. Clinical trials including regorafenib, another multi-kinase inhibitor (B-RAF, receptor tyrosine kinases (e.g., VEGFR, PDGFR, KIT) with antitumoral effects in preclinical studies [12,13], showed that the SMI may contribute to a survival benefit in recurrent glioblastoma [14,15,16], while a small clinical study based on six patients had a less beneficial outcome [17].

Another SMI is ipatasertib, a novel ATP-competitive AKT inhibitor, whose effects on glioblastoma cells have hardly been studied so far [18,19]. However, encouraging results were published in other solid tumors. In patients suffering from metastatic castration-resistant prostate cancer, ipatasertib was used in a chemotherapy regimen that contributed to a better tumor control in a patient cohort with an overall poor prognosis [20]. With respect to inoperable locally advanced triple-negative breast cancer, addition of ipatasertib to paclitaxel could help to prolong survival [21].

In the past, successful therapy of SMI in preclinical models of glioblastoma could not often be transferred to human studies [22,23]. It is believed that various mechanisms, such as a poor blood–brain/brain-tumor barrier permeability of the SMI and the development of resistance to a targeted therapy due to chronic administration, led to the generally disappointing results in clinical trials, which in the end limited the benefit of these targeted drugs in glioblastoma [24,25]. Therefore, the design of SMI is continuously improved to overcome defense mechanisms of the blood–brain/brain-tumor barriers in a sufficient extent, and in addition, agents that weaken tight junctions could improve the bioavailability of the SMI [25].

One possibility to improve the outcome could consist of personalized medicine therapy approaches [26]. Selecting patients who are potentially more likely to benefit from a therapy with SMI could result in a significant contribution to chemotherapy regimens for this cohort while avoiding unnecessary side effects for patients who are less likely to benefit. For this purpose, patient-derived tumor cells are increasingly acknowledged as a feasible tool to study the susceptibility to chemotherapeutics in vitro that interfere with signaling pathways [27,28,29]. In the current study, we took advantage of low-passage patient-derived glioblastoma cells [30]. As previously shown, these glioblastoma models were able to be used to study drug effects in preclinical and translational research [31,32,33].

Here, we systematically examined the effects of ipatasertib on patient-derived glioblastoma cells and additionally compared the results with four other SMIs addressing targets in the PI3K/AKT/mTOR and MAPK signaling pathway with different mechanisms of action. Furthermore, to investigate the impact of ipatasertib on the interaction of glioblastoma cells with the tumor-surrounding brain parenchyma, organotypic brain slices were established.

## 2. Materials and Methods

### 2.1. Patient-Derived Glioblastoma Cell Lines

In this study, four glioblastoma (WHO grade IV) low-passage cell lines were used as biological tumor model. All procedures were approved by the Ethics Committee of the Rostock University Medical Center (reference ID: A 2009/34) in accordance with generally accepted guidelines for the use of human material. Tumors were obtained from surgery, with informed written patient consent. The establishment of the glioblastoma cell lines (referred to as HROG cell lines) from primary brain tumor specimens and molecular characterization was described in detail in Mullins et al., 2013 [30]. Glioblastoma cell lines HROG02, HROG15, and HROG24 were established from untreated primary tumors, while the HROG05 cell line was obtained from a relapsed primary glioblastoma (Table 1).

All cell lines were cultured in Dulbecco’s Modified Eagle Medium: Nutrient Mixture F-12 (DMEM/F12; from PAN Biotech, Aidenbach, Germany) with 10% fetal calf serum (FCS, Bio&SELL, Feucht, Germany). Culturing the cells was performed at 37 °C in a 5% CO_2_ humidified atmosphere. All experiments were performed with ≤50 cell passages. At constant intervals, cell culture supernatants were tested for mycoplasma contamination employing MycoSPY^®^-PCR Mycoplasma Test Kit (Biontex, Munich, Germany).

### 2.2. Small-Molecule Kinase Inhibitors

All five small-molecule kinase inhibitors (SMI) were purchased from Selleck Chemicals (Houston, TX, USA) and, except for dactolisib (Dimethylformamide), were solved in dimethyl sulfoxide (DMSO) and stored at −80 °C for further use. In the culturing experiments, solvent volume fraction never exceeded 0.6%. A scheme illustrating the molecular targets of MK-2206 (allosteric AKT inhibitor), ipatasertib (ATP-competitive AKT inhibitor), dactolisib (PI3K/mTOR inhibitor), regorafenib (multi-kinase inhibitor), and MEK inhibitor trametinib is given in Supplementary Figure S1.

### 2.3. Quantification of DNA Synthesis

**Figure 1:**
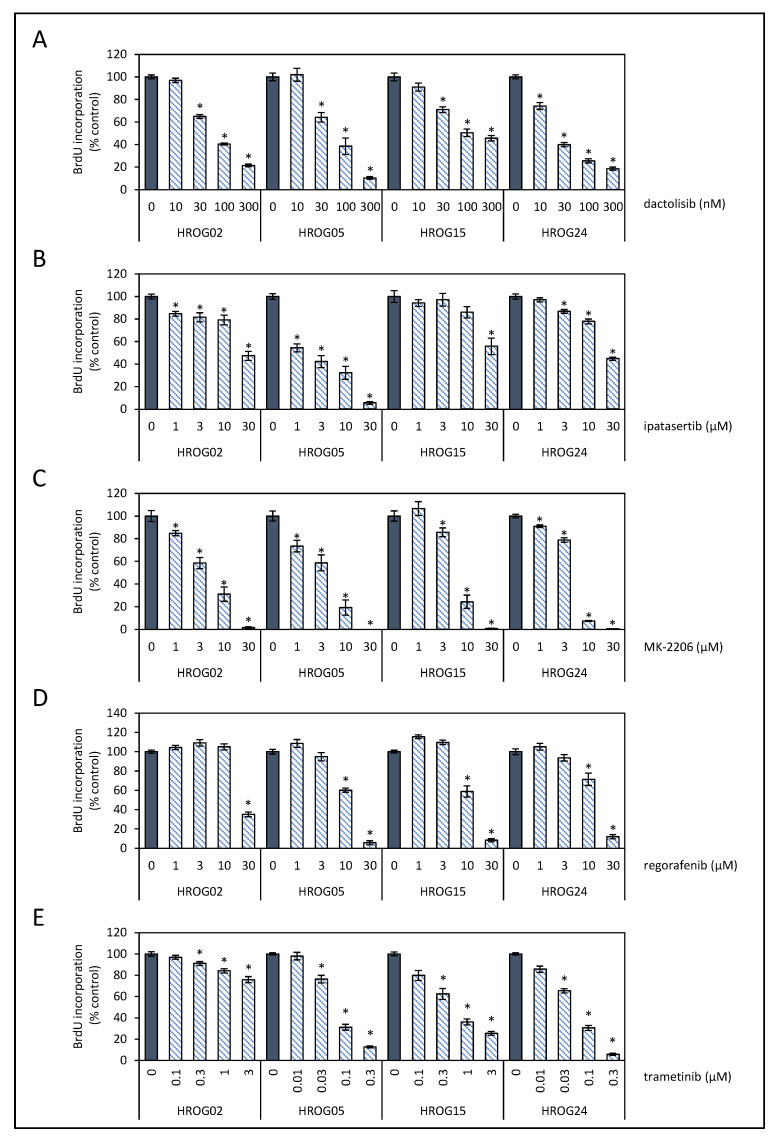
Effects of SMI on cell proliferation of glioblastoma cell lines. Patient-derived low-passage glioblastoma cell lines (HROG02, HROG05, HROG15, and HROG24) growing in 96-well half-area microplates were treated with (**A**) dactolisib, (**B**) ipatasertib, (**C**) MK-2206, (**D**) regorafenib, and (**E**) trametinib or solvent for 48 h, before DNA synthesis was measured with a BrdU incorporation assay. One hundred percent BrdU incorporation corresponds to cells cultured with the solvent only. Data are presented as mean ± SEM (*n* ≥ 12 separate cultures); * *p* < 0.05 versus control cultures (Kruskal–Wallis test with post hoc Dunn’s test).

To analyze effects of the SMI on DNA synthesis, a 5-bromo-2′-deoxyuridine (BrdU) incorporation assay kit (Roche Diagnostics GmbH, Mannheim, Germany) was used. Therefore, the cells were seeded in 96-half-area microplates at equal seeding densities (1 × 10^3^ cells/well) and allowed to adhere overnight in complete culture medium. On the following day, the culture medium was substituted with medium supplemented with the SMI at the indicated doses (Figure 1). After an incubation period of 32 h, BrdU labeling was initiated by adding labeling solution at a final concentration of 10 µM. Another 16 h later, labeling was stopped, and BrdU uptake was measured according to the manufacturer’s instructions using a GloMax-Multi Detection System (Promega, Madison, WI, USA).

### 2.4. Caspase 3/7 Activation Assay

**Figure 2:**
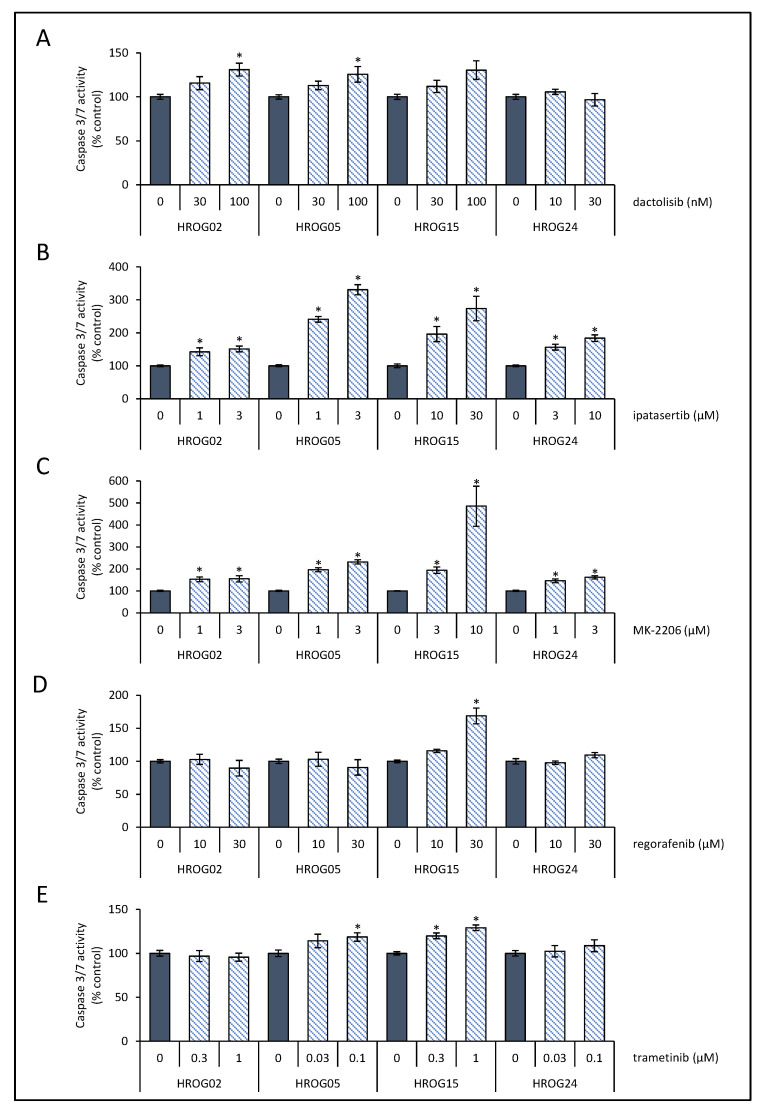
Effects of SMI on caspase 3/7 activity of glioblastoma cell lines. Glioblastoma cells were challenged with (**A**) dactolisib, (**B**) ipatasertib, (**C**) MK-2206, (**D**) regorafenib, (**E**) trametinib, or solvent control for 6 h. Afterwards, the cells were subjected to caspase 3/7 enzyme activity quantification. Data are presented as mean ± SEM (*n* ≥ 8 separate cultures for caspase activity assay); * *p* < 0.05 versus control cultures (Kruskal–Wallis test with post hoc Dunn’s test).

To gauge the effects of SMI on cell death by apoptosis, caspase 3/7 activity as surrogate marker was measured in a cell-based assay based on the luminogenic substrate Z-DEVD-aminoluciferin with a tetrapeptide sequence specific for caspase-3/7. Therefore, cells were seeded in 96-well plates (3 × 10^3^ cells/well) in complete culture medium. On the next day, either an inhibitor or the solvent was added at the indicated doses (Figure 2), and the incubation was continued for an additional period of 6 h. Afterwards, caspase activity was measured following the instructions of the manufacturer (Promega). Briefly, the cell culture plates were removed from the incubator, and the luminogenic substrate was added. Assay plates were incubated at 22 °C for 1 h before recording the luminescence with a GloMax microplate reader. For the generation of caspase 3/7-positive control cultures, cells were treated with 1 μM staurosporine (Sigma-Aldrich, Taufkirchen, Germany) for 2 h, and caspase activity was determined as described above (Supplementary Figure S2).

### 2.5. Immunoblotting

Glioblastoma cells were seeded in 12-well plates (3 × 10^4^ cells/well) in a complete culture medium. After two days, the cells were treated with SMI for 6 h. Then, protein extracts were prepared and subjected to immunoblot analysis as described before [34]. To receive total cellular protein, boiling lysis buffer (2% sodium dodecyl sulphate (SDS), 10% glycerol, 5 mM ethylenediaminetetraacetic acid (pH 8.0), 62.5 mM Tris-HCl (pH 6.8), 0.01% 3,3′,5,5′-tetrabromophenolsulfonphthalein, and 5% 2-mercaptoethanol) was added directly to the cell monolayer. Cellular proteins received from equal numbers of cells were separated by 10% SDS-polyacrylamide gel electrophoresis and blotted onto polyvinylidene fluoride (PVDF) membrane. Afterwards, membranes were blocked for 1 h using blocking buffer (10 mM Na_2_HPO_4_, 137 mM NaCl, 2 mM KH_2_PO_4_, 2.68 mM KCl, 0.05% Tween^®^ 20 (pH 7.4), and 2% bovine serum albumin (Carl Roth, Karlsruhe, Germany), before primary antibodies were added, and incubation continued overnight at 4 °C. All primary antibodies were from New England BioLabs (Frankfurt, Germany) unless specified otherwise: anti-phospho-AKT (P-AKT; #4060), anti-AKT protein (#4691), anti-phospho-ERK1/2 (P-ERK1/2) (#4370), anti-GAPDH (#2118), and anti-ERK1/2 (Abcam, Cambridge, UK; ab184699). The blots were developed using LI-COR reagents for an Odyssey Infrared Imaging System. The signal intensities of the investigated proteins were quantified by means of the Odyssey^®^ software 3.16 (LI-COR Biotechnology, Lincoln, NE, USA) and Image Studio Lite software 5.0 (LI-COR Biotechnology). Signals obtained for P-AKT, AKT protein, P-ERK1/2, and total ERK1/2 proteins were normalized for loading differences by calculating the ratio to GAPDH (total cellular protein). In a second step, signal intensities of P-AKT and P-ERK1/2 were normalized to total AKT and total ERK1/2 protein, respectively. Sample blots for each cell line and all inhibitors used in this study are presented in Supplementary Figures S3 and S4. Scans of entire PVDF membranes containing HROG24 lysates are shown in Supplementary Figure S7.

### 2.6. Organotypic Brain Slice Preparation

To establish cultures of organotypic brain slices, Fischer 344 rats (Charles River, Sulzfeld, Germany) were used as donors. All procedures were conducted according to national and international guidelines on the ethical use of animals (European Council Directive 86/609/EEC, approval of local authority LALLF (7221.3-2.4-006/12). All efforts were made to minimize animal suffering and to reduce the number of animals used. The animals were housed under environmentally controlled conditions (12 h light/dark cycles, lights switched on from 6 a.m. to 6 p.m., and 40–60% relative humidity).

Rats at the age of 6–8 days were deeply anesthetized by isoflurane inhalation (AbbVie, North Chicago, IL, USA) and decapitated. The brain was quickly removed and transferred into chilled and oxygenated (95% O_2_/5% CO_2_) dissection solution containing (in mmol/L) 87 NaCl, 25 NaHCO_3_, 2.5 KCl, 1.25 NaH_2_PO_4_, 0.5 CaCl_2_, 7 MgCl_2_, 10 D-glucose, and 75 sucrose adjusted to pH 7.4 with an osmolarity of 326–328 mosmol/l H_2_O. Next, the cerebellum was removed, and the brain was sectioned (350 µm coronal slices) on a vibratome (Integraslice 7550 MM, Campden Instruments Ltd., Loughborough, UK) in chilled and oxygenated artificial cerebrospinal fluid (aCSF). ACSF contained (in mmol/L): 124 NaCl, 26 NaHCO_3_, 3 KCl, 1.25 NaH_2_PO_4_, 2.5 CaCl_2_, 1.5 MgCl_2_, and 10 D-glucose adjusted to pH 7.4 with an osmolarity of 304–312 mosmol/L H_2_O. After preparation, slices were transferred onto Millicell, 6-well cell culture inserts (0.4 µm pore size, Merck Millipore) and cultured in slice culture medium (composed of 49% MEM with GlutaMAX (ThermoFisher Scientific, Waltham, MA, USA), 1% penicillin/streptomycin (PAN Biotech), 12.5% Basal Medium Eagle (BME), 12% FCS, and 0.5% glucose) as described [35].

### 2.7. HROG05 Glioblastoma on Organotypic Brain Slices

To distinguish tumor cells from the surrounding tissue of the brain slices, HROG05 cells were stained with carboxyfluorescein diacetate succinimidyl ester (CFDA) by employing Vybrant™ CFDA SE Cell Tracer Kit (ThermoFisher Scientific, Waltham, MA, USA). For this purpose, subconfluently growing HROG05 cells were incubated for 5 min in 10 µM CFDA solution (based on 10 mM stock solution diluted in phosphate-buffered saline), and afterwards, tumor cells were cultured for additional 24–72 h in complete culture medium. On the day of organotypic brain slice preparation, HROG05 cells were harvested, and 4 × 10^3^ cells/1 µL were placed onto the slices in cortical areas.

To analyze the effects of ipatasertib on HROG05 tumor growth and neuronal network activity, culture medium containing the AKT inhibitor or vehicle (DMSO) was replaced daily. Tumor size was estimated by quantification of the area of CFDA-bearing cells on the slices at 517 nm by laser-scanning microscopy (Leica DMI 6000, Wetzlar, Germany) employing Leica Application Suite (v. 2.0.0.13332) software (Leica, Wetzlar, Germany).

### 2.8. Field Potential Recordings

For electrophysiological recordings, slices were transferred into an interface chamber (BSC-HT, Harvard Apparatus, Holliston, MA, USA) maintained at 32 °C (TC-10, npi electronic GmbH, Tamm, Germany) and superfused with aCSF (perfusion rate of 2–3 mL/min). Under visual control, aCSF-filled glass micropipette field potential electrodes (Ag/AgCl with a resistance of approx. 2–5 MΩ) were placed 500–1000 µm from HROG05 glioblastoma above in neocortical layers. Field potentials were amplified, filtered at 1 kHz by an EXT-10–2F (npi electronic GmbH), and digitized using a Micro1401 analog-to-digital converter (Cambridge Electronic Design, Cambridge, UK) run by the Signal 2.16 software (Cambridge Electronic Design).

To evoke spontaneous rhythmic network activity in the brain slices, the slices were exposed to a well-established solution based on aCSF with 8 mM KCl, 0 mM MgCl_2_, and 5 µM gabazine (Tocris, Bristol, UK) [36]. Spontaneous rhythmic discharges were defined as field potential changes with an amplitude ≥2× noise amplitude. The frequency of these potentials was determined for the last 10 min after 2 h of exposure to the exhibition solution.

In initial pilot experiments, the network activity of slices without HROG05 tumors was estimated up to 14 days after slice preparation by quantifying spontaneous network deflections. The network activity rapidly decreased from day 1 to day 3 but afterwards remained relatively stable until day 10 (Supplementary Figure S5). We found a weak but significant correlation of days in culture and network deflections (*n* = 128; Pearson correlation coefficient was −0.435; *p* < 0.001). Therefore, for the experiments including HROG05 cells, the experimental period was limited up to seven days after slice preparation.

### 2.9. Statistical Analysis

Statistical analysis was performed with SigmaPlot 13.0. Experimental results are presented as mean ± standard error of the mean (SEM) for the indicated number of experiments. Mean group differences were tested for significance using the nonparametric Kruskal–Wallis test before for multiple comparisons; subgroups were tested with post hoc Dunn’s test. Comparisons of two independent groups were performed with a Mann–Whitney U test. To analyze effects of ipatasertib on organotypic brain slices, a three-way ANOVA followed by a Bonferroni *t*-test was performed. A two-way ANOVA followed by a Bonferroni *t*-test was conducted to test effects of ipatasertib on cell proliferation. A significance level of *p* < 0.05 was considered to be statistically significant.

## 3. Results

### 3.1. Effects on Glioblastoma Cell Growth of SMI Targeting Key Players of the PI3K/AKT/mTOR and MAPK Pathways

To study the antitumor efficacy of SMI that interfere in the PI3K/AKT/mTOR and MAPK signaling pathways, four low-passage cell lines of glioblastoma were employed. First, glioblastoma cells were exposed to different doses of multi-kinase inhibitor dactolisib (PI3K, mTOR), pan-AKT inhibitors ipatasertib and MK-2206, multi-kinase inhibitor regorafenib (e.g., various receptor tyrosine kinases, B-RAF), or MEK inhibitor trametinib, respectively. Cell proliferation was assessed by measuring the incorporation of BrdU into newly synthesized DNA (presented in alphabetical order in Figure 1). Inhibitor doses ranged from therapeutic to supra-therapeutic levels in patients’ sera as reported elsewhere [37,38,39,40,41]. Based on these data half-maximal effective concentration of all five tested inhibitors were calculated (Table 2).

Inhibitory effects of dactolisib on cell proliferation were detected in all glioblastoma cell lines, with HROG24 (EC_50_ = 24.1 nM) as the most sensitive to treatment in vitro model (Figure 1A; Table 2). As for the other three cell lines, EC_50_ values of 72.8–117.1 nM were determined. The AKT inhibitor MK-2206 attenuated DNA synthesis in all four glioblastoma cell lines with similar efficiency (EC_50_ = 4.6–7.1 µM, Figure 1C, Table 2).

In contrast, anti-proliferative effects of ipatasertib in low doses were primarily detected in HROG05 cell cultures (EC_50_ =1.7 µM), whereas that in HROG02 and HROG24 cells (EC_50_ = 28.4 µM and 26.9 µM, respectively) were less susceptible (Figure 1B, Table 2). Only at a high dose of ipatasertib (30 µM), DNA synthesis in HROG15 was found to be reduced, but no EC_50_ with the selected doses could be estimated. Furthermore, a two-way ANOVA (factor1: cell line, i.e., HROG02, HROG05, HROG15, and HROG24; factor 2: dose of ipatasertib, i.e., 0–30 µM) with Bonferroni post hoc test revealed that the proliferation of HROG05 glioblastoma cells was significantly more impaired by ipatasertib than in all other cell lines (*p* < 0.05; two-way ANOVA followed by Bonferroni *t*-test).

With respect to the SMI that interfere with key players of the MAPK pathway, regorafenib mediated similar inhibitory effects on all cell lines (EC_50_ = 13.5–25.7 µM; Figure 1D, Table 2). The MEK inhibitor trametinib reduced proliferation of HROG05 and HROG24 cells at low concentrations (EC_50_ = 72.1 nM and 62.6 nM, respectively), while the two other cell lines HROG02 and HROG15 were less susceptible (Figure 1E, Table 2).

### 3.2. Induction of Cell Death by SMI

Next, we investigated whether anti-proliferative effects on the glioblastoma cells were associated with cell death by apoptosis. As a surrogate marker for apoptosis, enzyme activity of caspases 3 and 7 was determined. Therefore, for each inhibitor, two concentrations (based on the BrdU incorporation data) were selected. As shown in Figure 2A, dactolisib slightly but significantly increased caspase 3/7 activity at 100 nM in HROG02 and HROG05, whereas HROG15 and HROG24 were unaffected at the selected doses. Both AKT inhibitors significantly increased caspase activity in all four cell lines (Figure 2B,C). After incubation with regorafenib, only in HROG15, an increase in caspase 3/7 activity was determined (Figure 2D). As shown in Figure 2E, in HROG05 and HROG15 an increased caspase activity was found after incubation with trametinib, whereas HROG02 and HROG24 were unaffected (Figure 2E).

### 3.3. Effects of SMI on PI3K/AKT/mTOR and MAPK Signaling in Glioblastoma Cells

**Figure 3:**
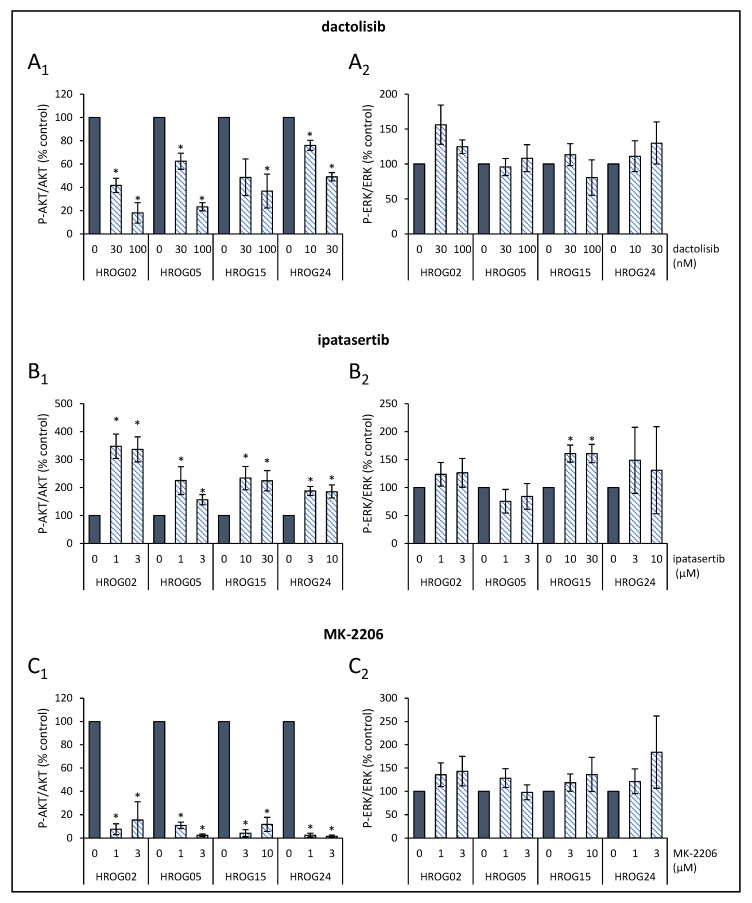
Quantitative analysis of the effects of dactolisib, ipatasertib and MK-2206 on signal transduction in HROG cell lines. The effects of (**A_1_**,**A_2_**) dactolisib, (**B_1_**,**B_2_**) ipatasertib, and (**C_1_**,**C_2_**) MK-2206 on fluorescence signal intensities of phosphoproteins (P-AKT and P-ERK1/2, respectively) and corresponding total proteins in HROG02, HROG05, HROG15, and HROG24 cells were quantified. Subsequently, the ratios P-AKT/AKT protein and P-ERK/ERK protein were determined. Data of 5–7 independent experiments were used to calculate mean values ± SEM, * *p* < 0.05 versus control cultures (Mann–Whitney U test).

**Figure 4:**
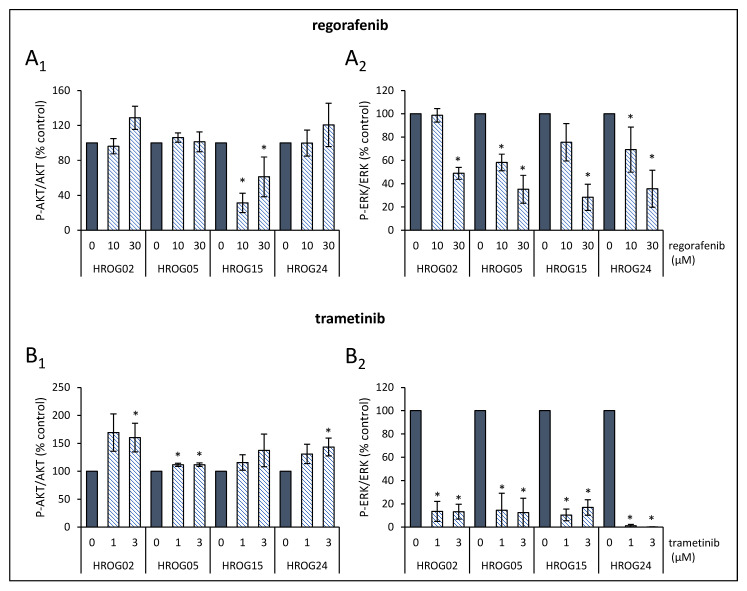
Effects of regorafenib and trametinib on signal transduction in HROG cell lines. The effects of (**A_1_**,**A_2_**) regorafenib and (**B_1_**,**B_2_**) trametinib on phosphoprotein levels of AKT and ERK1/2 and corresponding total proteins in HROG02, HROG05, HROG15, and HROG24 cells were quantified. Subsequently, the ratios P-AKT/AKT protein and P-ERK/ERK protein were determined. Data of 5–7 independent experiments were used to calculate mean values ± SEM, * *p* < 0.05 versus control cultures (Mann–Whitney U test).

We next studied how the investigated SMI affected the phosphorylation of AKT and ERK1/2 in HROG02, HROG05, HROG15, and HROG24 cells. For each cell line, representative immunoblots are shown in Supplementary Figures S3 and S4, while the quantitative effects of all five SMI are presented in Figure 3 and Figure 4.

In agreement with the biological efficacy, dactolisib diminished AKT phosphorylation in a dose-dependent manner (Figure 3(A_1_)), whereas MK-2206 to a large extent abolished the Phospho-AKT level (Figure 3(C_1_)). No effects on Phospho-ERK1/2 level following exposure to dactolisib or MK-2206 were detected (Figure 3(A_2_,C_2_)). In marked contrast, ipatasertib significantly increased phosphorylation of AKT on residue serine 473 in all four cell lines (Figure 3(B_1_)), which reflects the mechanism of action of the ATP-competitive inhibitor [42]. This could be seen as an indicator that ipatasertib successfully bound to AKT, which is associated with its biological effects on cell proliferation and apoptosis. Furthermore, in HROG15 cells, Phospho-ERK1/2 levels were elevated after treatment with ipatasertib Figure 3(B_2_).

Multi-kinase inhibitor regorafenib attenuated phospho-ERK1/2 level in all cell lines in a dose-dependent manner (Figure 4(A_2_)). With respect to the PI3K/AKT/mTOR pathways, no effect on phosphorylation of AKT was detected except in HROG15 cell cultures that presented an attenuated phosphorylation of the kinase (Figure 4(A_1_)). As a highly specific MEK1/2 inhibitor, trametinib abolished ERK1/2 phosphorylation (Figure 4(B_2_)). At the same time, there was an increase in the phospho-AKT level in all cell lines in a significant (HROG02, HROG05, and HROG24) and non-significant (HROG15) manner (Figure 4(B_1_)).

### 3.4. Ipatasertib Attenuates HROG05 Glioblastoma Growth on Organotypic Brain Slices

Since ipatasertib presented a great potential to inhibit proliferation of HROG05 glioblastoma cells in vitro, we next focused on the question whether these effects could be translated to a co-culture model of glioblastoma cells growing on organotypic brain slices, a viable model between cell culture studies and in vivo animal studies with orthotopic growing tumors [35,43]. Based on preliminary experiments, slices were analyzed up to 7 days after preparation, as in vital slices, this period was found to be robust to detect sufficient synaptic activity within the cortical network (Supplementary Figure S5).

**Figure 5:**
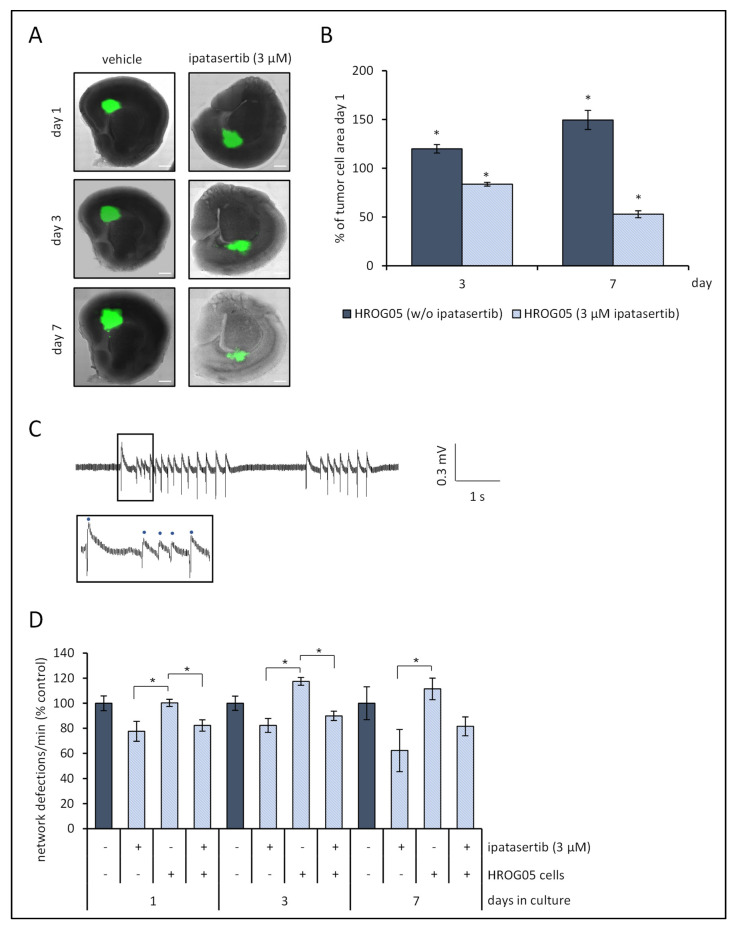
Ipatasertib inhibits HROG05 glioblastoma tumor growth and diminishes network activity of organotypic slice cultures. Organotypic brain slices (350 µm) of Fischer 344 rats at the age of 6–8 days were prepared, and 4 × 10^3^ HROG05 cells were placed onto the slices on day of preparation. In addition, slices w/o HROG05 glioblastoma cells were prepared for control studies. The next day, vehicle or ipatasertib (3 µM) were added to culture medium and renewed in a daily routine. (**A**) Representative overlay photographs based on bright-field and fluorescence images of slices bearing HROG glioblastoma ± ipatasertib (3 µM) illustrate HROG05 cell growth (scale bar = 1 mm). (**B**) Quantification of HROG05 tumor size. Data represent mean values ± SEM as percent of day 1 (= start of ipatasertib treatment) to estimate the effects of solvent (DMSO) or SMI on each slice separately, *n* = 11–12 number of slices per group, * *p* < 0.05 versus day 1 tumor size (Kruskal–Wallis test followed by post hoc analysis (Dunn’s test)). (**C**) The slices were exposed to aCSF with 8 mM KCl, 0 mM Mg^2+^, and 5 mM gabazine, and field potential recordings were performed in surrounding cortical area. A sample trace of field potential recordings of day 3 is presented. Dots above sample trace indicate network deflections that were used for quantitative analysis. (**D**) Quantification of network deflections of the last 10 min after 2 h incubation in aCSF with 8 mM KCl, 0 mM Mg^2+^, and 5 mM gabazine. The data are presented as percent of network deflections of vehicle-treated slices for each day, *n* = 13–30 number of measurements, * *p* < 0.05 versus control cultures of the same day (Kruskal–Wallis test followed by post hoc Dunn’s test).

With respect to tumor growth, untreated HROG05 glioblastoma expanded onto organotypic brain slices up to 149.6 ± 9.8% on day 7 in comparison to the tumor area of day 1 (Figure 5A,B), while the addition of ipatasertib (3 µM) into the culture medium attenuated the growth of HROG24 tumor down to 52.9 ± 3.5% during the same period. To test effects of ipatasertib on the slices themselves, propidium iodide uptake of the cells as surrogate marker for membrane integrity and cell death was estimated. The selected dose of 3 µM of the AKT inhibitor had no effect on propidium iodide uptake (Supplementary Figure S6).

Furthermore, we investigated the impact of (a) HROG05 glioblastoma and (b) the treatment with ipatasertib on the network activity. To this purpose, spontaneous rhythmic network discharges in slices exposed to aCSF with 8 mM KCl, 0 mM MgCl_2_, and 5 µM gabazine were quantified. As shown in pilot experiments, the number of network discharges decreased over the course of the cultivation period (Supplementary Figure S5B). This was also evident for the DMSO control group (*n* = 67; Pearson correlation coefficient for factor day and network deflections: −0.75, *p* < 0.001). Therefore, to analyze the effects of glioblastoma and exposure to ipatasertib, network deflections were normalized to vehicle-treated cultures of the same day (Figure 5C,D).

On day 1 and day 3, slices with ipatasertib (w/ or w/o HROG05) presented with a lower amount of network deflections than slices w/HROG05 of the same day, whereas on day 7, network discharges were attenuated by ipatasertib in comparison to slices with tumor cells only (*p* < 0.05, Kruskal–Wallis test with post hoc Dunn’s test).

Additionally, a three-way ANOVA (factor day, i.e., 1, 3, and 7, factor treatment, i.e., ipatasertib versus vehicle control, and factor HROG05 glioblastoma versus slices w/o tumor cells) with Bonferroni post hoc *t*-test revealed that the AKT inhibitor diminished network deflections regardless of the absence or presence of glioblastoma cells (*p* < 0.001). As expected, a daily decrease in the network activity could be observed. The presence of HROG05 cells increased overall network activity in comparison to slices without tumor cells (*p* = 0.01). Noteworthy, the effect of ipatasertib was not significantly correlated to the day of measurement (*p* = 0.356).

## 4. Discussion

Despite intensive efforts during the last decades by experimental and clinical groups, survival of patients diagnosed with glioblastoma is often limited to a few months. Since the establishment of temozolomide as an add-on to radiotherapy, no other drug could be identified as part of a standard chemotherapy regimen. Due to the frequency of alterations in the PI3K/AKT/mTOR and MAPK signaling pathways, SMI that target key players in the pathway may contribute to a survival benefit for selected patients at this point [24,44].

The allosteric AKT inhibitor MK-2206 was found to mediate antitumoral effects on permanent cell lines [8,9,45,46]. Indeed, we also found an overall uniform response to MK-2206 in all patient-derived cell lines in a dose range comparable to published references [8,46]. Interestingly, Djuzenova et al. (2019) showed that glioblastoma cells could be sensitized to an irradiation regimen with complementary MK-2206 medication, but this seemed to be limited to PTEN-wildtype tumor cells [9]. Moreover, Savill et al. (2022) showed that acquired resistance to MK-2206 can be overcome with complementary ipatasertib [47].

Unlike MK-2206, the dual PI3K/mTOR inhibitor dactolisib presented with a less uniform response pattern with respect to cell proliferation. In contrast to ipatasertib and MK-2206, this compound led only at high concentrations to a mild but significant induction of apoptosis. Notably, Shi et al., (2018) found a proportion of 20% of the glioblastoma cells underwent apoptosis, but at the same time, a huge proportion of the cells was arrested at the G0/G1 phase of cell cycle [6]. In prostate and breast cancer cells expressing PTEN-wildtype, dactolisib induced cell death, while cells with loss-of-function mutations or deletions of PTEN were much more resistant [48,49]. In our study, there was no correlation between PTEN status and response to dactolisib.

The multi-kinase inhibitor regorafenib effectively abolished glioblastoma cell growth at concentrations in the range of 10–30 µM, leading to similar EC_50_ in all cell lines. Interestingly, in comparison to astrocytes, permanent glioblastoma cell lines were found to be much more susceptible to a regorafenib treatment in vitro [13]. While U87 cells had an IC_50_ of 17 µM and U251 an IC_50_ of 12 µM, in human astrocytes (NHA), at clinically relevant doses, no IC_50_ was determined [13]. Our data indicate that apoptosis is not the principal mechanism of action in the tested glioblastoma models. Instead, overactivation of autophagy via stabilization of phosphoserine aminotransferase 1 (PSAT1) may contribute to the antitumoral effect of regorafenib [13]. However, results presented by Chiang et al., (2022) showed a proapoptotic profile of the SMI [50]. Of all SMI that were investigated, regorafenib was the only drug that currently had contributed to first promising results in the treatment of glioblastoma. In patients suffering from recurrent glioblastoma, a randomized phase II trial (named REGOMA) revealed that a therapy based on the multi-kinase inhibitor led to a prolonged progression-free period and an overall longer survival [14]. A subsequent analysis identified biomarkers that were associated with response to regorafenib, which may help to select patients that could benefit most from the SMI [51]. Eventually, a phase III study is required to finally assess the efficacy of regorafenib in therapy regimens of glioblastoma.

In three of four glioblastoma cell lines, we found antitumoral effects of the MEK inhibitor trametinib. In line with previous studies, our investigation came out with comparable EC_50_ values [10,52,53]. Currently, trametinib was used in combination with dabrafenib (B-Raf inhibitor) in glioma harboring BRAF^V600E^ mutation [11,54,55,56,57]. BRAF^V600E^ alterations rarely occur in glioblastoma, but for this specific patient cohort, the targeted therapy could be potentially beneficial. While the case studies reported mixed results of the combination regimen in different types of brain cancer, the ROAR study focusing on high-grade glioma is currently under investigation [11].

For the first time, we showed that the AKT inhibitor ipatasertib attenuated patient-derived glioblastoma cell growth in vitro and in a co-culture model on organotypic brain slices. In all investigated HROG cell lines, incubation with ipatasertib led to cell death via apoptosis. In colorectal cancer cells, Sun et al., (2018) found that the apoptosome-mediated intrinsic way of apoptosis was an important mechanism of action of ipatasertib [58]. This is in line with our findings that caspases 3 and 7, which are downstream of caspase 9 in the cascade of caspases, were found to be highly activated after AKT inhibitor treatment. In agreement with the molecular mechanism of ATP-competitive AKT inhibitors [42,59], the treatment of the cells with ipatasertib led to an increase in the phosphorylation level of the kinase to lock the enzyme in an activated but non-functional state.

Remarkably, HROG05 was found to be highly susceptible to ipatasertib, while the other cell lines were only affected at high, supra-therapeutic concentrations of this AKT inhibitor. HROG05 cells are the only patient-derived glioblastoma model in our study that harbors a mutation in the protooncogene KRAS (G12D), a G protein that drives the activation of downstream pathways. A G12D mutation was associated with an impaired hydrolysis of GTP to GDP on the G protein, which ended in a constitutive activation of KRAS [60]. One may speculate that inhibition of the PI3K/AKT/mTOR pathway may particularly also affect the KRAS-driven MAPK pathway, as both pathways are linked via crosstalk [61]. However, exposure of the HROG cells to MK-2206 resulted in a uniform response pattern to the allosteric inhibitor.

Another finding was that ipatasertib diminished the network activity in organotypic brain slices independently of the presence of glioblastoma cells. It can be assumed that the synchronous network discharges are dependent on glutamatergic synaptic transmission. The influx of Na^+^ and Ca^2+^ via ionotropic glutamate receptors contributes to the generation of excitatory postsynaptic potentials, and eventually the generation of action potentials (via voltage-dependent sodium channels) in post-synaptic neurons. Therefore, glutamate receptor antagonists were established as effective drugs to treat glioma-associated epilepsy [62]. In addition, the PI3K/AKT/mTOR pathway was identified as a molecular target to control spontaneous seizure in a preclinical model of epilepsy [63]. Kim et al. found a correlation of GluA1 (an AMPA receptor subunit) expression and seizure control after AKT inhibition. As symptomatic hyperexcitability in the form of tumor-associated epilepsy is frequently reported in patients with glioblastoma [64], the additional attenuating effect of ipatasertib on the network activity may be of clinical relevance in an add-on chemotherapy setting. Our data indicate that ipatasertib may be worth testing in further in vivo studies and probably also in clinical trials. However, a limiting factor for a persistent effectiveness of a targeted therapy is acquired resistance to the SMI. Recently, it was demonstrated that acquired resistance to ipatasertib could be overcome with the addition of Moloney-murine leukemia virus (PIM) kinase inhibitors, as it was demonstrated that PIM kinases are highly involved in the resistance to ATP-competitive AKT inhibitors like ipatasertib [47].

In our study, we used organotypic brain slices as a co-culture model to investigate the impact of ipatasertib not only on the tumor cells themselves but also on the glioblastoma-surrounding tissue. With respect to the 3R principle (replacement, reduction, and refinement), organotypic brain slices can be a valuable link between cell culture experiments (2D/3D) and animal tumor model. However, several disadvantages, such as the exclusion of the immune system and a relative short maximal observation period (1–3 weeks), limit the usefulness of this in vitro model [34,65].

Overall, our study sheds light on the effects of inhibitors that interfere with the mostly affected signaling pathways in glioblastoma. While especially the multi-kinase inhibitor regorafenib and both MK-2206 and dactolisib were very effective in all preclinical tumor models, ipatasertib showed fewer beneficial effects. Our data indicate that potentially only a subset of patients may benefit from a therapy including ipatasertib. Therefore, further in vivo studies based on orthotopically growing glioblastoma cells should address different mutation patterns and additionally should not disregard the anti-excitatory potential of ipatasertib.

## 5. Conclusions

In conclusion, in patient-derived cell lines of glioblastoma, we demonstrated for the first time that ipatasertib exhibits anti-tumoral effects in vitro and in a co-culture model of organotypic brain slices. Here, HROG05, a glioblastoma model harboring a KRAS mutation, turned out to be highly susceptible, while other cell lines were resistant to low concentrations of the AKT inhibitor. Our data on four additional SMI support previous findings on glioblastoma cells in vitro and in vivo. Importantly, ipatasertib had no pro-apoptotic effect on healthy brain parenchyma but attenuated the activity of the neuronal network in the brain slices.

## Figures and Tables

**Table 1:** Patient-derived glioblastoma models.

Tumor ID	Gender/Age	Mutations	Tumor Species (Origin)
HROG02	M/68	TP53 R248Q	Primary glioblastoma
HROG05	F/60	KRAS G12D PTEN P169S/del 212–229	Relapsed primary glioblastoma
HROG15	M/56	TP53 R273H PTEN S170N	Primary glioblastoma
HROG24	F/73	TP53 R273C PTEN exon 3 del/spliced	Primary glioblastoma

**Table 2:** Potency of SMI based on BrdU incorporation assay (EC_50_).

	Dactolisib (nM)	Ipatasertib (µM)	MK-2206 (µM)	Regorafenib (µM)	Trametinib (nM)
HROG02	72.8	28.3	5.2	25.7	#
HROG05	68.6	1.7	4.6	13.7	72.1
HROG15	117.1	#	7.1	13.5	631.4
HROG24	24.1	26.9	5.8	17.2	62.6

# no inhibitory effects on BrdU incorporation under experimental conditions.

## Data Availability

The data presented in this study are available on request from the corresponding author.

## Supplementary Materials

The following supporting information can be downloaded at: https://www.mdpi.com/article/10.3390/life12081258/s1, Figure S1: Small-molecule kinase inhibitors address targets within the MAPK and PI3K/AKT/mTOR pathway and receptor tyrosine kinases; Figure S2: Effects of staurosporine on caspase 3/7 activity in glioblastoma cells; Figure S3: Effects of dactolisib, ipatasertib and MK-2206 on the phosphorylation of AKT and ERK1/2 in glioblastoma cell lines; Figure S4: Effects of regorafenib and trametinib on the phosphorylation of AKT and ERK1/2 in glioblastoma cell lines; Figure S5: Characterization of organotypic brain slices; Figure S6: Effect of ipatasertib on organotypic brain slices of Fischer 344 rats; Figure S7: Sample PVDF membranes of SMI effects on AKT and ERK1/2 activation in HROG24 cell cultures; Table S1: Signal intensities from protein bands quantified in immunoblot analyses; Table S2: Signal intensities from protein bands quantified in immunoblot analyses.

## References

[1] Ostrom Q.T., Gittleman H., Farah P., Ondracek A., Chen Y., Wolinsky Y., Stroup N.E., Kruchko C., Barnholtz-Sloan J.S. (2013). CBTRUS statistical report: Primary brain and central nervous system tumors diagnosed in the United States in 2006–2010. Neuro-Oncology.

[2] Verhaak R.G.W., Hoadley K.A., Purdom E., Wang V., Qi Y., Wilkerson M.D., Miller C.R., Ding L., Golub T., Mesirov J.P. (2010). Cancer Genome Atlas Research Network. Integrated genomic analysis identifies clinically relevant subtypes of glioblastoma characterized by abnormalities in PDGFRA, IDH1, EGFR, and NF1. Cancer Cell.

[3] Zhang P., Xia Q., Liu L., Li S., Dong L. (2020). Current Opinion on Molecular Characterization for GBM Classification in Guiding Clinical Diagnosis, Prognosis, and Therapy. Front. Mol. Biosci..

[4] Brennan C.W., Verhaak R.G.W., McKenna A., Campos B., Noushmehr H., Salama S.R., Zheng S., Chakravarty D., Sanborn J.Z., Berman S.H. (2013). TCGA Research Network. The somatic genomic landscape of glioblastoma. Cell.

[5] Nakada M., Kita D., Watanabe T., Hayashi Y., Teng L., Pyko I.V., Hamada J.-I. (2011). Aberrant signaling pathways in glioma. Cancers.

[6] Shi F., Zhang J., Liu H., Wu L., Jiang H., Wu Q., Liu T., Lou M., Wu H. (2017). The dual PI3K/mTOR inhibitor dactolisib elicits anti-tumor activity in vitro and in vivo. Oncotarget.

[7] Netland I.A., Førde H.E., Sleire L., Leiss L., Rahman M.A., Skeie B.S., Gjerde C.H., Enger P.Ø., Goplen D. (2016). Dactolisib (NVP-BEZ235) toxicity in murine brain tumour models. BMC Cancer.

[8] Narayan R.S., Fedrigo C.A., Brands E., Dik R., Stalpers L.J.A., Baumert B.G., Slotman B.J., Westerman B.A., Peters G.J., Sminia P. (2017). The allosteric AKT inhibitor MK2206 shows a synergistic interaction with chemotherapy and radiotherapy in glioblastoma spheroid cultures. BMC Cancer.

[9] Djuzenova C.S., Fiedler V., Memmel S., Katzer A., Sisario D., Brosch P.K., Göhrung A., Frister S., Zimmermann H., Flentje M. (2019). Differential effects of the Akt inhibitor MK-2206 on migration and radiation sensitivity of glioblastoma cells. BMC Cancer.

[10] Selvasaravanan K.D., Wiederspohn N., Hadzalic A., Strobel H., Payer C., Schuster A., Karpel-Massler G., Siegelin M.D., Halatsch M.-E., Debatin K.-M. (2020). The limitations of targeting MEK signalling in Glioblastoma therapy. Sci. Rep..

[11] Wen P.Y., Stein A., van den Bent M., De Greve J., Wick A., de Vos F.Y.L., von Bubnoff N., van Linde M.E., Lai A., Prager G.W. (2022). Dabrafenib plus trametinib in patients with BRAFV600E-mutant low-grade and high-grade glioma (ROAR): A multicentre, open-label, single-arm, phase 2, basket trial. Lancet Oncol..

[12] Wilhelm S.M., Dumas J., Adnane L., Lynch M., Carter C.A., Schütz G., Thierauch K.-H., Zopf D. (2011). Regorafenib (BAY 73-4506): A new oral multikinase inhibitor of angiogenic, stromal and oncogenic receptor tyrosine kinases with potent preclinical antitumor activity. Int. J. Cancer.

[13] Jiang J., Zhang L., Chen H., Lei Y., Zhang T., Wang Y., Jin P., Lan J., Zhou L., Huang Z. (2020). Regorafenib induces lethal autophagy arrest by stabilizing PSAT1 in glioblastoma. Autophagy.

[14] Lombardi G., De Salvo G.L., Brandes A.A., Eoli M., Rudà R., Faedi M., Lolli I., Pace A., Daniele B., Pasqualetti F. (2019). Regorafenib compared with lomustine in patients with relapsed glioblastoma (REGOMA): A multicentre, open-label, randomised, controlled, phase 2 trial. Lancet Oncol..

[15] Lombardi G., Caccese M., Padovan M., Cerretti G., Pintacuda G., Manara R., Di Sarra F., Zagonel V. (2021). Regorafenib in Recurrent Glioblastoma Patients: A Large and Monocentric Real-Life Study. Cancers.

[16] Detti B., Scoccianti S., Lucidi S., Maragna V., Teriaca M.A., Ganovelli M., Desideri I., Lorenzetti V., Scoccimarro E., Greto D. (2021). Regorafenib in glioblastoma recurrence: A case report. Cancer Treat. Res. Commun..

[17] Kebir S., Rauschenbach L., Radbruch A., Lazaridis L., Schmidt T., Stoppek A.-K., Pierscianek D., Stuschke M., Forsting M., Sure U. (2019). Regorafenib in patients with recurrent high-grade astrocytoma. J. Cancer Res. Clin. Oncol..

[18] Lin J., Sampath D., Nannini M.A., Lee B.B., Degtyarev M., Oeh J., Savage H., Guan Z., Hong R., Kassees R. (2013). Targeting activated Akt with GDC-0068, a novel selective Akt inhibitor that is efficacious in multiple tumor models. Clin. Cancer Res..

[19] Yan Y., Serra V., Prudkin L., Scaltriti M., Murli S., Rodríguez O., Guzman M., Sampath D., Nannini M., Xiao Y. (2013). Evaluation and Clinical Analyses of Downstream Targets of the Akt Inhibitor GDC-0068. Clin. Cancer Res..

[20] Sweeney C., Bracarda S., Sternberg C.N., Chi K.N., Olmos D., Sandhu S., Massard C., Matsubara N., Alekseev B., Parnis F. (2021). Ipatasertib plus abiraterone and prednisolone in metastatic castration-resistant prostate cancer (IPATential150): A multicentre, randomised, double-blind, phase 3 trial. Lancet.

[21] Dent R., Oliveira M., Isakoff S.J., Im S.-A., Espié M., Blau S., Tan A.R., Saura C., Wongchenko M.J., Xu N. (2021). LOTUS investigators Final results of the double-blind placebo-controlled randomized phase 2 LOTUS trial of first-line ipatasertib plus paclitaxel for inoperable locally advanced/metastatic triple-negative breast cancer. Breast Cancer Res. Treat..

[22] Yang K., Wu Z., Zhang H., Zhang N., Wu W., Wang Z., Dai Z., Zhang X., Zhang L., Peng Y. (2022). Glioma targeted therapy: Insight into future of molecular approaches. Mol. Cancer.

[23] Jain K.K. (2018). A Critical Overview of Targeted Therapies for Glioblastoma. Front. Oncol..

[24] Colardo M., Segatto M., Di Bartolomeo S. (2021). Targeting RTK-PI3K-mTOR Axis in Gliomas: An Update. Int. J. Mol. Sci..

[25] Wang D., Wang C., Wang L., Chen Y. (2019). A comprehensive review in improving delivery of small-molecule chemotherapeutic agents overcoming the blood-brain/brain tumor barriers for glioblastoma treatment. Drug Deliv..

[26] Darrigues E., Elberson B.W., De Loose A., Lee M.P., Green E., Benton A.M., Sink L.G., Scott H., Gokden M., Day J.D. (2021). Brain Tumor Biobank Development for Precision Medicine: Role of the Neurosurgeon. Front. Oncol..

[27] Johansson P., Krona C., Kundu S., Doroszko M., Baskaran S., Schmidt L., Vinel C., Almstedt E., Elgendy R., Elfineh L. (2020). A Patient-Derived Cell Atlas Informs Precision Targeting of Glioblastoma. Cell Rep..

[28] Jacob F., Ming G.L., Song H. (2020). Generation and biobanking of patient-derived glioblastoma organoids and their application in CAR T cell testing. Nat. Protoc..

[29] Stringer B.W., Day B.W., D’Souza R.C.J., Jamieson P.R., Ensbey K.S., Bruce Z.C., Lim Y.C., Goasdoué K., Offenhäuser C., Akgül S. (2019). A reference collection of patient-derived cell line and xenograft models of proneural, classical and mesenchymal glioblastoma. Sci. Rep..

[30] Mullins C.S., Schneider B., Stockhammer F., Krohn M., Classen C.F., Linnebacher M. (2013). Establishment and characterization of primary glioblastoma cell lines from fresh and frozen material: A detailed comparison. PLoS ONE.

[31] Lange F., Weßlau K., Porath K., Hörnschemeyer J., Bergner C., Krause B.J., Mullins C.S., Linnebacher M., Köhling R., Timo Kirschstein T. (2019). AMPA receptor antagonist perampanel affects glioblastoma cell growth and glutamate release in vitro. PLoS ONE.

[32] Clancy H., Pruski M., Lang B., Ching J., McCaig C.D. (2021). Glioblastoma cell migration is directed by electrical signals. Exp. Cell Res..

[33] Xie Y., Bergström T., Jiang Y., Johansson P., Marinescu V.D., Lindberg N., Segerman A., Wicher G., Niklasson M., Baskaran S. (2015). The Human Glioblastoma Cell Culture Resource: Validated Cell Models Representing All Molecular Subtypes. EBioMedicine.

[34] Lange F., Franz B., Maletzki C., Linnebacher M., Hühns M., Jaster R. (2014). Biological and molecular effects of small molecule kinase inhibitors on low-passage human colorectal cancer cell lines. Biomed Res. Int..

[35] Lange F., Hörnschemeyer J., Kirschstein T. (2021). Glutamatergic Mechanisms in Glioblastoma and Tumor-Associated Epilepsy. Cells.

[36] Lange F., Hartung J., Liebelt C., Boisserée J., Resch T., Porath K., Hörnschemeyer J., Reichart G., Sellmann T., Neubert V. (2020). Perampanel Add-on to Standard Radiochemotherapy in vivo Promotes Neuroprotection in a Rodent F98 Glioma Model. Front. Neurosci..

[37] Infante J.R., Fecher L.A., Falchook G.S., Nallapareddy S., Gordon M.S., Becerra C., DeMarini D.J., Cox D.S., Xu Y., Morris S.R. (2012). Safety, pharmacokinetic, pharmacodynamic, and efficacy data for the oral MEK inhibitor trametinib: A phase 1 dose-escalation trial. Lancet Oncol..

[38] Saura C., Roda D., Roselló S., Oliveira M., Macarulla T., Pérez-Fidalgo J.A., Morales-Barrera R., Sanchis-García J.M., Musib L., Budha N. (2017). A First-in-Human Phase I Study of the ATP-Competitive AKT Inhibitor Ipatasertib Demonstrates Robust and Safe Targeting of AKT in Patients with Solid Tumors. Cancer Discov..

[39] Doi T., Tamura K., Tanabe Y., Yonemori K., Yoshino T., Fuse N., Kodaira M., Bando H., Noguchi K., Shimamoto T. (2015). Phase 1 pharmacokinetic study of the oral pan-AKT inhibitor MK-2206 in Japanese patients with advanced solid tumors. Cancer Chemother. Pharmacol..

[40] Wise-Draper T.M., Moorthy G., Salkeni M.A., Karim N.A., Thomas H.E., Mercer C.A., Beg M.S., O’Gara S., Olowokure O., Fathallah H. (2017). A Phase Ib Study of the Dual PI3K/mTOR Inhibitor Dactolisib (BEZ235) Combined with Everolimus in Patients with Advanced Solid Malignancies. Target. Oncol..

[41] Kobayashi K., Sugiyama E., Shinozaki E., Wakatsuki T., Tajima M., Kidokoro H., Aoyama T., Nakano Y., Kawakami K., Hashimoto K. (2021). Associations among plasma concentrations of regorafenib and its metabolites, adverse events, and ABCG2 polymorphisms in patients with metastatic colorectal cancers. Cancer Chemother. Pharmacol..

[42] Lazaro G., Kostaras E., Vivanco I. (2020). Inhibitors in AKTion: ATP-competitive vs allosteric. Biochem. Soc. Trans..

[43] Savaskan N.E., Seufert S., Hauke J., Tränkle C., Eyüpoglu I.Y., Hahnen E. (2011). Dissection of mitogenic and neurodegenerative actions of cystine and glutamate in malignant gliomas. Oncogene.

[44] Hannen R., Hauswald M., Bartsch J.W. (2017). A Rationale for Targeting Extracellular Regulated Kinases ERK1 and ERK2 in Glioblastoma. J. Neuropathol. Exp. Neurol..

[45] Cheng Y., Zhang Y., Zhang L., Ren X., Huber-Keener K.J., Liu X., Zhou L., Liao J., Keihack H., Yan L. (2012). MK-2206, a novel allosteric inhibitor of Akt, synergizes with gefitinib against malignant glioma via modulating both autophagy and apoptosis. Mol. Cancer Ther..

[46] Jin R., Nakada M., Teng L., Furuta T., Sabit H., Hayashi Y., Demuth T., Hirao A., Sato H., Zhao G. (2013). Combination therapy using Notch and Akt inhibitors is effective for suppressing invasion but not proliferation in glioma cells. Neurosci. Lett..

[47] Savill K.M.Z., Lee B.B., Oeh J., Lin J., Lin E., Chung W.-J., Young A., Chen W., Miś M., Mesh K. (2022). Distinct resistance mechanisms arise to allosteric vs. ATP-competitive AKT inhibitors. Nat. Commun..

[48] Wang W., Long L., Yang N., Zhang Q., Ji W., Zhao J., Qin Z., Wang Z., Chen G., Liang Z. (2013). NVP-BEZ235, a novel dual PI3K/mTOR inhibitor, enhances the radiosensitivity of human glioma stem cells in vitro. Acta Pharmacol. Sin..

[49] Brachmann S.M., Hofmann I., Schnell C., Fritsch C., Wee S., Lane H., Wang S., Garcia-Echeverria C., Maira S.-M. (2009). Specific apoptosis induction by the dual PI3K/mTor inhibitor NVP-BEZ235 in HER2 amplified and PIK3CA mutant breast cancer cells. Proc. Natl. Acad. Sci. USA.

[50] Chiang I., Liu Y.-C., Liu H.-S., Ali A.A.A., Chou S.-Y., Hsu T., Hsu F.-T. (2022). Regorafenib Reverses Temozolomide-Induced CXCL12/CXCR4 Signaling and Triggers Apoptosis Mechanism in Glioblastoma. Neurotherapeutics.

[51] Santangelo A., Rossato M., Lombardi G., Benfatto S., Lavezzari D., De Salvo G.L., Indraccolo S., Dechecchi M.C., Prandini P., Gambari R. (2021). A molecular signature associated with prolonged survival in glioblastoma patients treated with regorafenib. Neuro-Oncology.

[52] Essien E.I., Hofer T.P., Atkinson M.J., Anastasov N. (2022). Combining HDAC and MEK Inhibitors with Radiation against Glioblastoma-Derived Spheres. Cells.

[53] Schreck K.C., Allen A.N., Wang J., Pratilas C.A. (2020). Combination MEK and mTOR inhibitor therapy is active in models of glioblastoma. Neurooncol. Adv..

[54] Fusco M.J., Piña Y., Macaulay R.J., Sahebjam S., Forsyth P.A., Peguero E., Walko C.M. (2021). Durable Progression-Free Survival With the Use of BRAF and MEK Inhibitors in Four Cases with BRAF V600E-Mutated Gliomas. Cancer Control..

[55] Brown N.F., Carter T., Kitchen N., Mulholland P. (2017). Dabrafenib and trametinib in BRAFV600E mutated glioma. CNS Oncol..

[56] Schreck K.C., Guajardo A., Lin D.D.M., Eberhart C.G., Grossman S.A. (2018). Concurrent BRAF/MEK Inhibitors in BRAF V600-Mutant High-Grade Primary Brain Tumors. J. Natl. Compr. Cancer Netw..

[57] Smith-Cohn M., Davidson C., Colman H., Cohen A.L. (2019). Challenges of targeting BRAF V600E mutations in adult primary brain tumor patients: A report of two cases. CNS Oncol..

[58] Sun L., Huang Y., Liu Y., Zhao Y., He X., Zhang L., Wang F., Zhang Y. (2018). Ipatasertib, a novel Akt inhibitor, induces transcription factor FoxO3a and NF-κB directly regulates PUMA-dependent apoptosis. Cell Death Dis..

[59] Lin K., Lin J., Wu W.I., Ballard J., Lee B.B., Gloor S.L., Vigers G.P.A., Morales T.H., Friedman L.S., Skelton N. (2012). An ATP-site on-off switch that restricts phosphatase accessibility of Akt. Sci. Signal..

[60] Hunter J.C., Manandhar A., Carrasco M.A., Gurbani D., Gondi S., Westover K.D. (2015). Biochemical and Structural Analysis of Common Cancer-Associated KRAS Mutations. Mol. Cancer Res..

[61] Sunayama J., Matsuda K.-I., Sato A., Tachibana K., Suzuki K., Narita Y., Shibui S., Sakurada K., Kayama T., Tomiyama A. (2010). Crosstalk between the PI3K/mTOR and MEK/ERK pathways involved in the maintenance of self-renewal and tumorigenicity of glioblastoma stem-like cells. Stem Cells.

[62] Huberfeld G., Vecht C.J. (2016). Seizures and gliomas—Towards a single therapeutic approach. Nat. Rev. Neurol..

[63] Kim J.E., Lee D.S., Park H., Kim T.H., Kang T.C. (2021). Inhibition of AKT/GSK3β/CREB Pathway Improves the Responsiveness to AMPA Receptor Antagonists by Regulating GRIA1 Surface Expression in Chronic Epilepsy Rats. Biomedicines.

[64] Kerkhof M., Dielemans J.C.M., van Breemen M.S., Zwinkels H., Walchenbach R., Taphoorn M.J., Vecht C.J. (2013). Effect of valproic acid on seizure control and on survival in patients with glioblastoma multiforme. Neuro-Oncology.

[65] Eisemann T., Costa B., Strelau J., Mittelbronn M., Angel P., Peterziel H. (2018). An advanced glioma cell invasion assay based on organotypic brain slice cultures. BMC Cancer.

