# Supplementary material for: Studies on Biological and Molecular Effects of Small-Molecule Kinase Inhibitors on Human Glioblastoma Cells and Organotypic Brain Slices
Source: Life (Basel). 2022 Aug 17;12(8):1258. doi: 10.3390/life12081258 (PMC9409734; doi:10.3390/life12081258)
Supplement: Supplementary file 1 [file life-12-01258-s001.zip › life-1845110-supplementary.pdf]

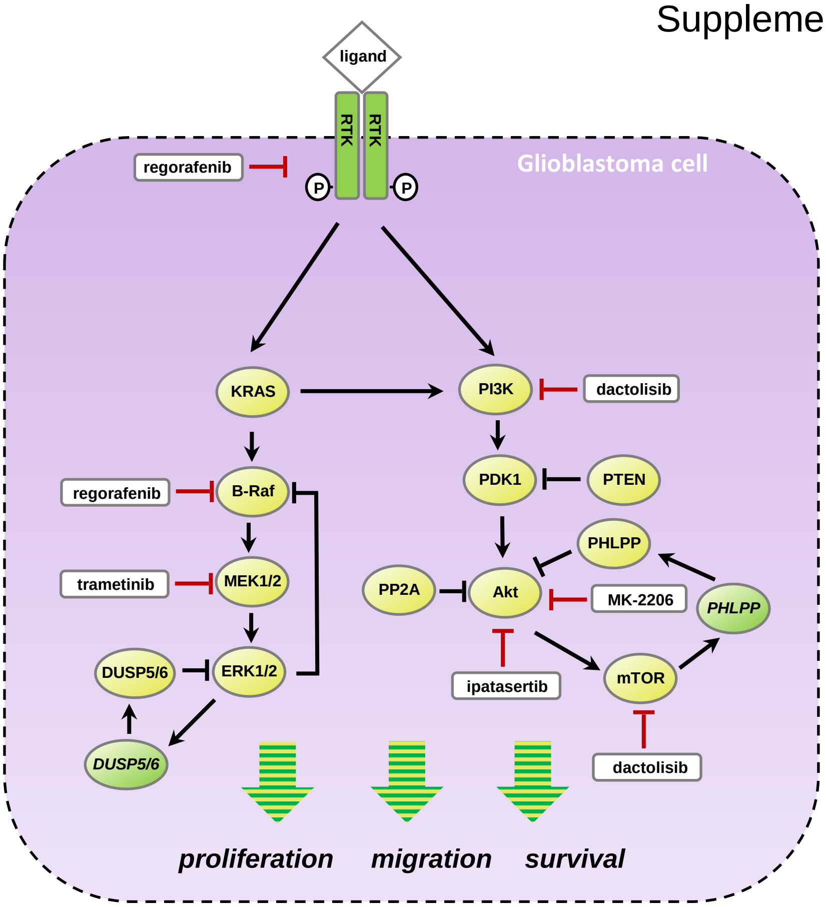

Supplementary Figure S1: Small-molecule kinase inhibitors address targets within the MAPK and PI3K/AKT/mTOR pathway and receptor tyrosine kinases. Abbrv.: Extracellular regulated kinase 1/2 (**ERK1/2**), dual-specificity phosphatase (**DUSP**), Kirsten rat sarcoma virus (**KRAS**), mitogen-activated protein kinase kinase 1/2 (**MEK1/2**), mammalian target of rapamycin (**mTOR**), phosphoinositide-dependent kinase-1 (**PDK1**), phosphoinositide 3-kinases (***PI3K***), PH domain and leucine rich repeat protein phosphatase (**PHLPP**), protein phosphatase 2 (**PP2A**), phosphatase and tensin homolog (**PTEN**), receptor tyrosine kinase (**RTK**)

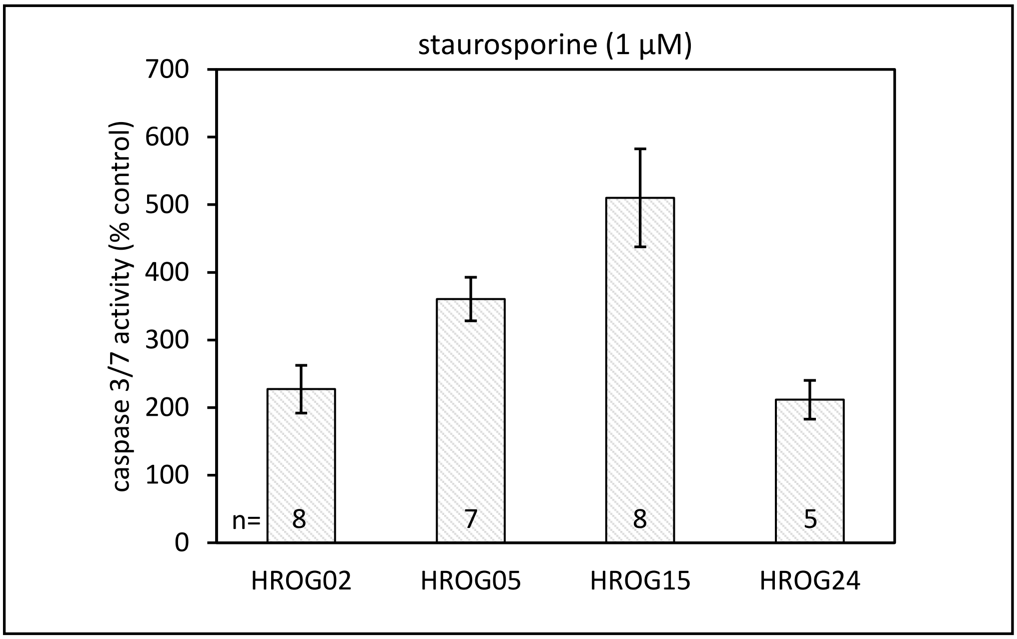

Supplementary Figure S2: Effects of staurosporine on caspase 3/7 activity in glioblastoma cells. Glioblastoma cells were challenged with staurosporine (1 µM) for 2 hours. Afterwards, caspase 3/7 activity was estimated as described in Materials and methods section. Data are presented as mean ± SEM for the indicated number (n) of biological replicates.

Supplementary Figure S3

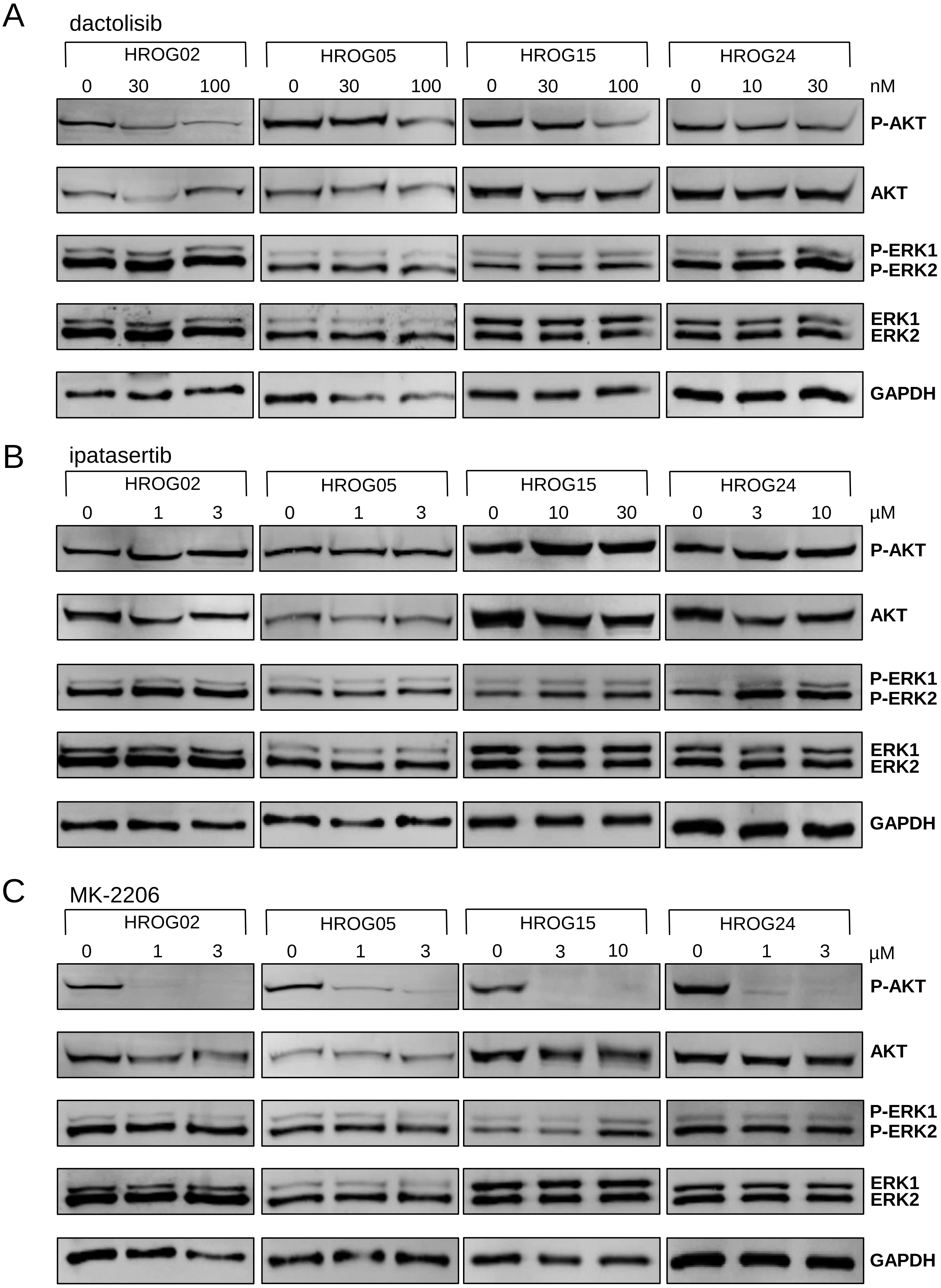

Supplementary Figure S3: Effects of dactolisib, ipatasertib and MK-2206 on the phosphorylation of AKT and ERK1/2 in glioblastoma cell lines. For each cell line, one representative blot is shown. For mean values of independent experiments, please refer to Figure 3.

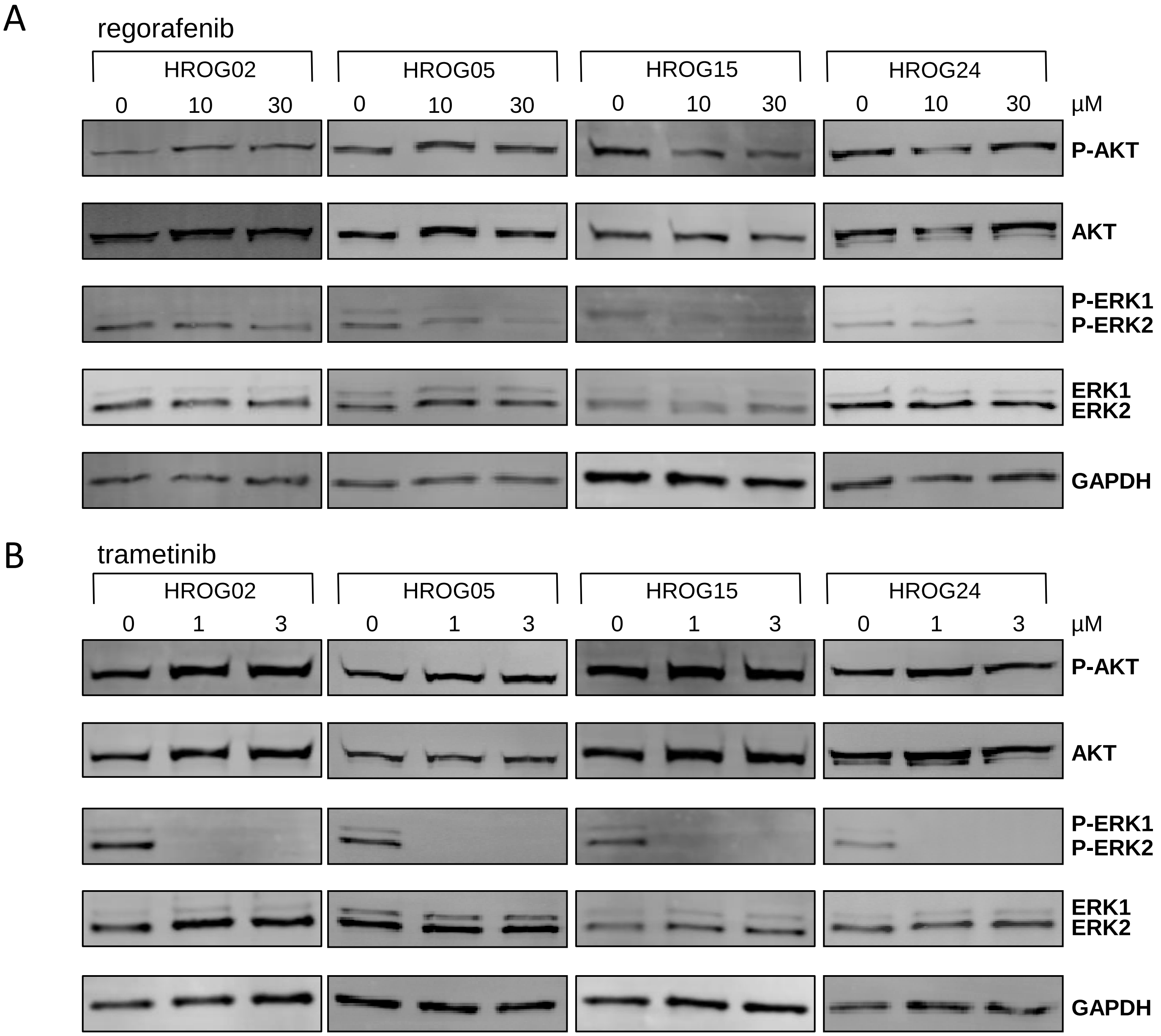

Supplementary Figure S4: Effects of regorafenib and trametinib on the phosphorylation of AKT and ERK1/2 in glioblastoma cell lines. For each cell line, one representative blot is shown. For mean values of independent experiments, please refer to Figure 4 in the manuscript.

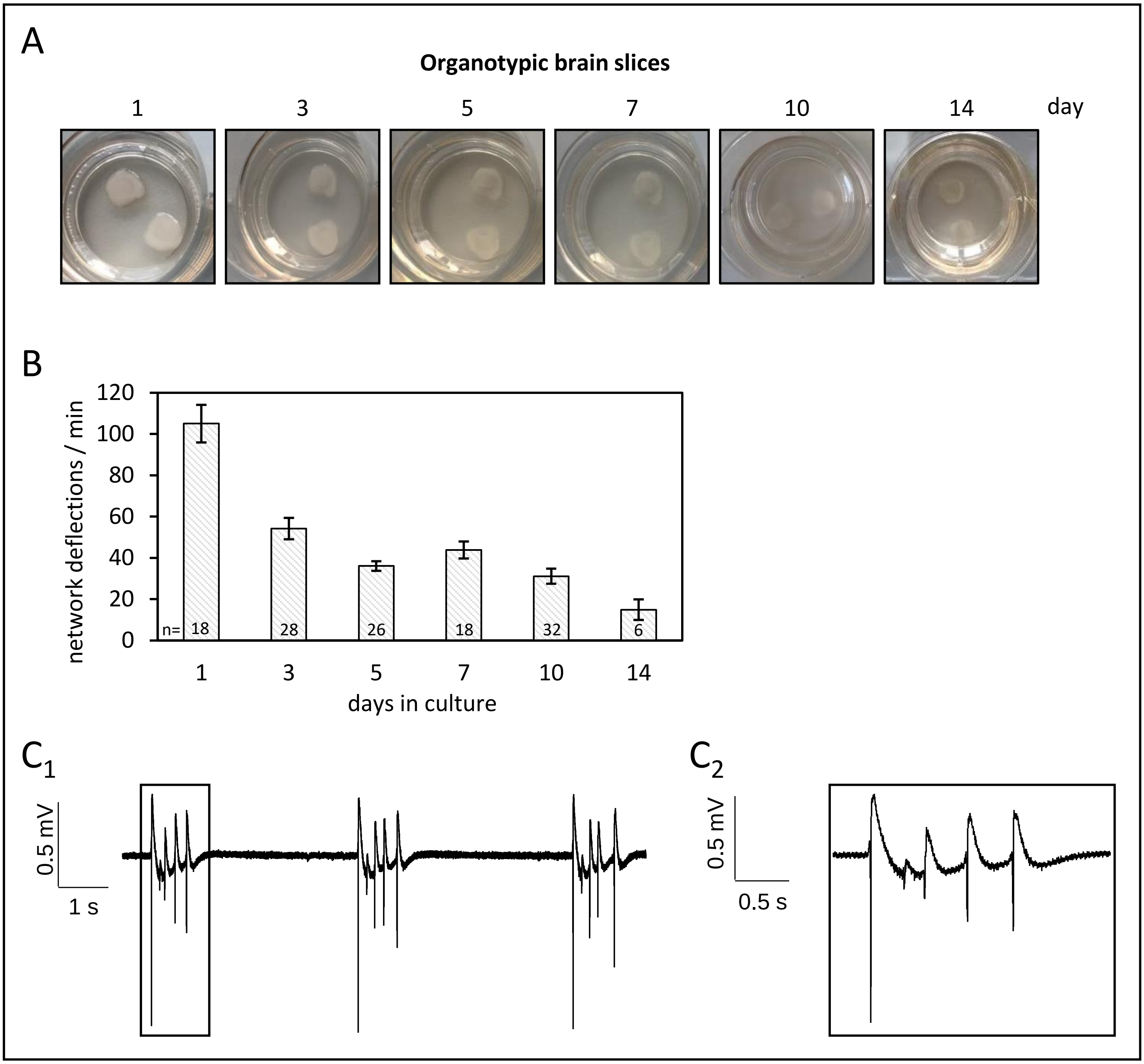

Supplementary Figure S5: Characterization of organotypic brain slices. Fischer 344 rats at the age of 6-8 days were sacrificed and coronal brain slices (350 µm) were prepared. (**A**) The slices were cultured up to 14 days in Millicell, 6-well cell culture Inserts. (**B**) At the indicated days, network deflections were electrophysiologically recorded in cortical areas. Data represent mean ± SEM, n=number of slices included in the analysis. (**C1,C2**) Representative sample trace of field potential recordings at day 3.

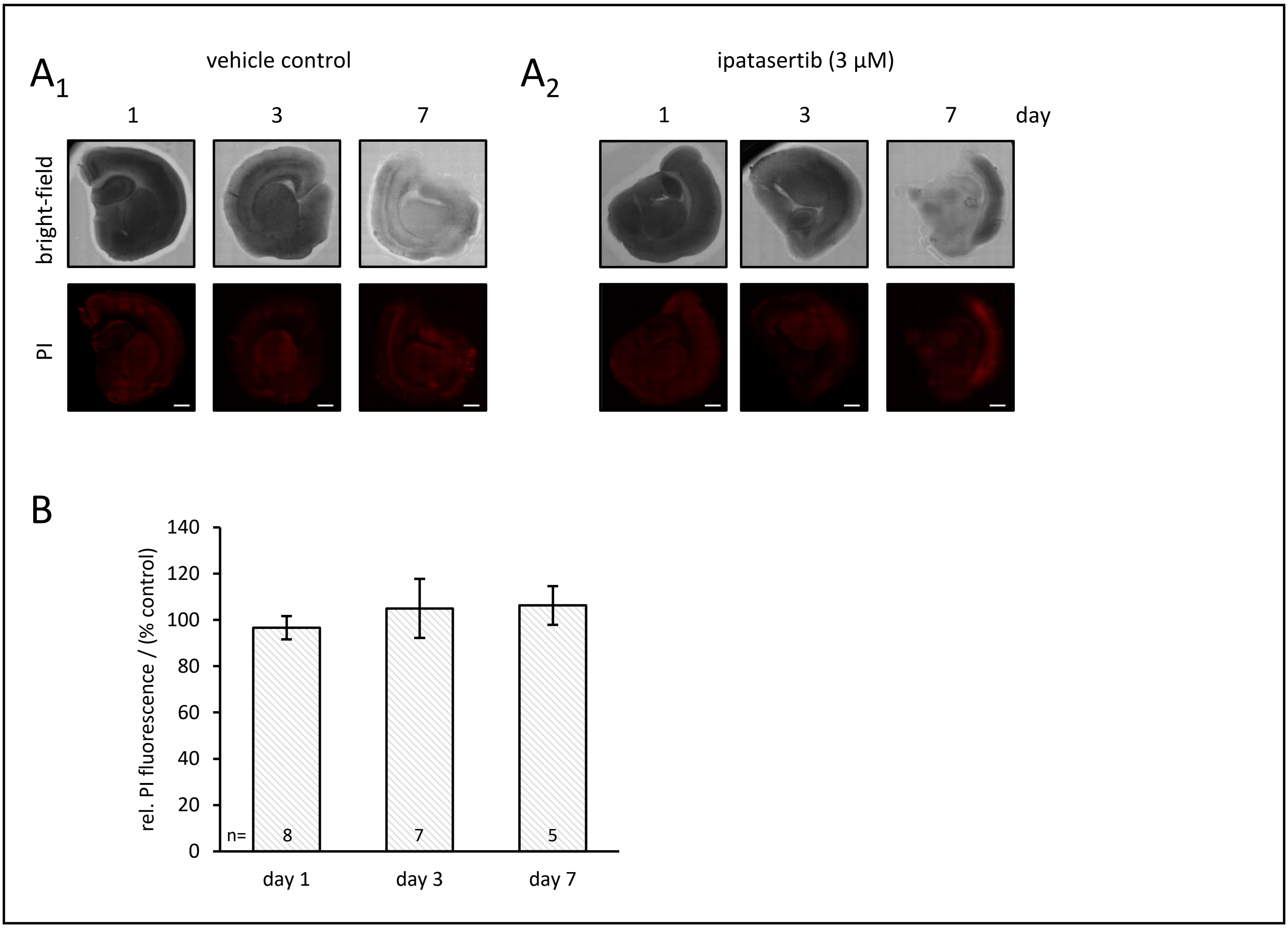

Supplementary Figure S6: Effect of ipatasertib on organotypic brain slices of Fischer 344 rats. Fischer rats at the age of 6-8 days were sacrificed and coronal brain slices (350 µm) were prepared. The slices were cultured in complete culture medium (see Materials and methods for details). Starting with the day of preparation, the slices were exposed to (**A$_1$**) vehicle or (**A$_2$**) ipatasertib (3 µM) for the indicated periods of time and stained with propidium iodide (PI) based on a standard protocol. Scale bar represents 1 mm.(**B**) Quantification of PI fluorescence. Data are represented as mean ± SEM of PI fluorescence in comparison to control slices w/o ipatasertib of the same day, n=number of biological replicates included in the analysis. No significant changes between control cultures and ipatasertib-treated cultures were found (U test).

Supplementary Figure S7: Sample PVDF membranes of SMI effects on AKT and ERK1/2 activation in HROG24 cell cultures. Precision Plus Protein Dual Color Standards (BIO-RAD) and Prestained Protein Standard, Broad Range (11-250 kDa) biomarker (New England Biolabs) were used as molecular size markers. For further details see Materials and Methods section in the manuscript.

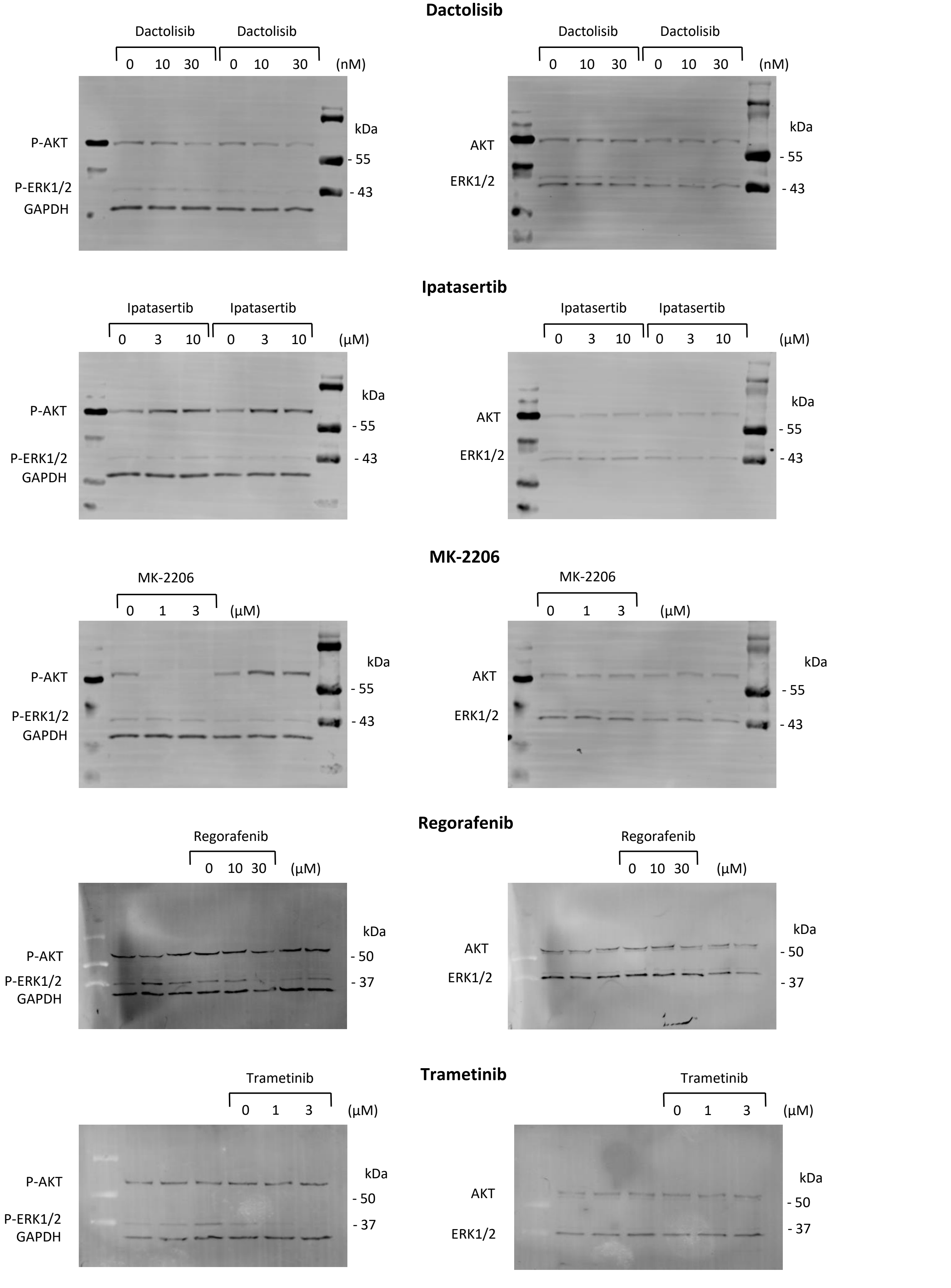

Table S1: Signal intensities from protein bands quantified in immunoblot analyses

		Dactolisib [nM]											
		HROG02			HROG05			HROG 15			HROG 24		
		0	30	100	0	30	100	0	30	100	0	10	30
repl. 1	P-Akt	0.67	0.49	0.39	5.72	2.99	0.89	5.78	1.43	0.03	2.81	2.37	1.42
	Akt	2.27	3.09	2.2	4.49	4.56	3.87	6.23	4.95	4.05	0.84	1.19	0.81
	P-Erk	2.36	0.92	2.03	2.66	2.17	2.67	2.71	3.7	1.96	0.65	0.89	0.71
	Erk	6.06	6.36	6.16	8.59	11.22	10.57	7.34	7.36	7.52	1.58	2.55	1.78
	GAPDH	6.35	7.43	5.94	8.13	7.42	6.03	9.85	9.5	9.98	5.24	5.71	4.48
repl. 2	P-Akt	0.4	0.09	0.05	4.92	1.88	0.84	3.27	1.42	0.21	3.14	2.32	1.51
	Akt	3.3	3.86	3.32	3.93	3.64	4.34	4.92	5.94	6.39	0.88	0.86	0.96
	P-Erk	1.6	2.37	1.98	2.34	2.68	1.65	2.29	1.28	1.07	0.45	0.49	0.34
	Erk	9.42	8.64	8.1	8.87	16.17	14.51	5.27	7.05	10.27	1.32	1.88	1.15
	GAPDH	7.09	8.67	8.88	5.66	8.11	8.34	8.99	9.79	9.48	5.87	5.73	5.99
repl. 3	P-Akt	3.75	0.5	0.24	15.44	6.7	1.88	6.83	0.61		4.43	3.66	1.95
	Akt	1	0.43	0.35	3.77	1.82	1.52	1.74	0.96		1.27	1.48	1.43
	P-Erk	2.77	2.92	2.04	3.15	2.82	2.38	3.11	2.71	0.61	1.19	0.68	0.67
	Erk	3.52	2.12	1.97	3.63	2.99	1.53	3.57	2.78	3.43	4.62	3.46	2.59
	GAPDH	7.55	6.48	7.31	11.69	7.88	8.16	8.71	4.84	4.34	11.86	8.56	9.26
repl. 4	P-Akt	5.89	1.78	0.05	10.43	4.5	1.65	7.42	1.78	2.91	3.5	1.89	1.29
	Akt	1.67	1.01	0.87	2.35	1.61	1.4	2.33	1.4	2.03	0.93	0.63	0.62
	P-Erk	2.41	4.95	3	2.11	2.8	2.92	2.78	3.57	3.72	0.23	0.28	0.13
	Erk	4.82	4.24	4.19	1.98	1.87	2.9	2.79	3.05	3.13	1.42	1.57	1.48
	GAPDH	11.37	11.37	7.73	8.18	6.83	6.88	6.66	6.55	8.1	7.72	6.86	7.26
repl. 5	P-Akt	1.43	0.42	0.02	1.79	0.42	0.12	1.15	0.43	0.2	2.13	2.19	1.24
	Akt	0.85	0.65	0.58	0.93	0.38	0.43	0.63	0.54	0.15	0.94	1.23	1.3
	P-Erk	1	2.14	1.33	1.41	1.6	1.13	2.53	2.9	1.02	0.41	0.44	0.46
	Erk	2.84	2.98	3.55	1.39	1.66	0.79	1.5	1.44	0.92	3.57	3.81	2
	GAPDH	9.58	6.66	7.47	1.92	1.82	2.2	2.81	3.68	2.75	5.66	5.5	5.88
repl. 6	P-Akt	1.9	1.5	0.3	3.32	1.87	0.84	1.34	1.19	0.54	1.78	1.4	1.1
	Akt	1.62	2.2	1.84	1.69	1.33	1.21	1.7	1.21	1.14	1.33	1.14	1.33
	P-Erk	3.43	4.23	3.83	1.37	1.35	1.18	2.03	2.57	2.89	0.93	1.72	2.17
	Erk	6.41	6.25	5.17	3.7	3.48	2.87	2.87	2.36	2.22	2.89	2.45	2.78
	GAPDH	6.47	6.66	5.74	4.08	4.54	3.95	3.73	3.39	3.55	2.99	2.72	3.06

		Ipatasertib [µM]											
		HROG02			HROG05			HROG 15			HROG 24		
		0	1	3	0	1	3	0	10	30	0	3	10
repl. 1	P-Akt	2.03	7.12	6.66	2.79	3.03	1.68	1.34	1.92	1.99	3.56	8.46	7.65
	Akt	2.19	1.83	2.02	1.08	0.91	0.41	0.79	0.63	0.4	1.36	2.17	2.21
	P-Erk	0.79	1.05	1.54	0.97	1.2	1.28	0.3	0.67	0.97	1.98	1.36	1.21
	Erk	0.95	1	1.41	0.23	0.46	0.32	2.77	3.14	5.06	4.77	3.39	3.97
	GAPDH	1.42	1.35	1.97	0.53	0.42	0.23	2.22	3.65	4.55	9.01	8.49	10.4
repl. 2	P-Akt	0.8	3.89	2.85	1.71	1.93	3.94				3.86	7.98	7.29
	Akt	1.45	1.54	1.3	0.58	0.35	0.85				2.12	2.31	2.69
	P-Erk	2.22	0.67	0.66	1.01	1.25	1.39	0.9	1.3	1.07	0.91	0.91	0.66
	Erk	3.65	1.15	1.57	0.14	0.35	0.54	6.09	5.06	4.77	3.11	1.03	3.87
	GAPDH	14.42	6.39	7.79	3.85	2.73	5.63	5.27	4.5	5.49	10.72	7.76	10.59
repl. 3	P-Akt	0.62	4.35	2.83	2.69	2.09	2.88	1.26	2.87	2.63	4.61	7.99	8.51
	Akt	0.28	1.27	0.92	1.06	0.43	0.88	1.08	1.44	1.5	1.68	1.67	1.52
	P-Erk	0.2	0.48	0.24	0.25		0.42	0.43	0.95	0.97	1.03	0.11	0.7
	Erk	2.49	2.93	1.37	0.89		1.03	1.29	1.6	1.43	3.73	3.38	4.66
	GAPDH	3.27	3.37	2.5	1.21	0.98	1.44	1.9	4.32	4.61	9.78	8.39	8.22
repl. 4	P-Akt	0.57	0.88	2.44	1.38	1.45	2.77	0.96	1.12	2.5	3.87	8.52	7.82
	Akt	0.87	0.35	0.8	0.35	0.08	0.59	1.16	0.53	1.53	1.25	1.43	1.16
	P-Erk	0.61	0.32	0.52	0.55	0.3	0.05	0.36	0.46	0.57	0.63	0.23	0.07
	Erk	1.82	1.41	2.96	0.29	0.6	0.67	1.43	1.6	2	3.4	1.4	0.88
	GAPDH	3.21	2.34	3.44	2.02	1.84	2.2	4.16	4.8	4.87	5.4	6.46	6.8
repl. 5	P-Akt	1.32	2.45	5.13	1.25	4.72	5.18	3.85	6.87	5.46	2.19	5.57	5.45
	Akt	1.21	0.63	1.4	0.48	0.76	0.82	1.42	1.46	1.31	0.82	1.33	1.53
	P-Erk	0.44	0.57	0.59	0.23	1.04	1.36	0.97	1.1	1.77	1.25	0.48	0.2
	Erk	5.57	8.76	6.77	1.99	6.03	8.27	1.28	1.29	2.01	2.91	3.23	1.27
	GAPDH	4.21	4.64	3.27	1.19	3.35	4.11	6.08	7.38	5.89	5.88	6	6.31
repl. 6	P-Akt	1.91	5.3	5.1	2.57	3.45	3.39	1.18	3.66	3.26	1.39	2.31	3.55
	Akt	2.28	2.04	1.89	1.36	1.3	1.37	1.88	1.5	1.59	1.35	0.86	1.25
	P-Erk	2.27	3.25	2.7	1.63	1.6	1.48	1.19	1.6	1.76	0.41	1.11	1.66
	Erk	8.94	7.77	6.04	3.77	4.14	4.14	3.84	2.75	2.82	3.5	2.67	2.73
	GAPDH	7.12	6.74	6.37	4.25	3.82	4.07	4.39	3.89	3.8	3.15	2.75	2.92

		MK-2206 [µM]											
		HROG02			HROG05			HROG 15			HROG 24		
		0	1	3	0	1	3	0	3	10	0	1	3
repl. 1	P-Akt	2.61	0	0	2	0.23	0	2.83	0.04	0.03	7.21	0	0
	Akt	1.18	0.72	0.04	0.46	0.64	0.38	1.02	0.98	0.79	2.25	3.15	2.09
	P-Erk	1.29	1.46	0.97	0.96	1.54	1.55	0.36	1.31	1.72	2.38	1.97	3.52
	Erk	0.62	0.77	0.19	0.31	0.24	0.29	3.48	6.1	6.05	3.47	3.36	3.45
	GAPDH	4.66	3.23	1.28	2.12	2.52	2.38	4.94	6.46	5.85	14.86	14.42	12.88
repl. 2	P-Akt	1.26	0.02	0	2.16	0.26	0.16	3.49	0.03	0.01	11.66	0.42	0
	Akt	0.78	0.61	0.4	0.61	0.54	0.69	0.86	0.89	0.84	3.89	4.33	3.68
	P-Erk	1.23	1.07	0.92	1.82	1.79	1.24	1.02	0.99	0.76	0.27	1.31	2.83
	Erk	0.7	0.66	0.68	0.48	0.28	0.3	3.52	3.77	3.7	4.71	8.96	8.54
	GAPDH	4.94	3.9	2.79	2.61	2.5	2.97	4.01	4.38	3.65	14.45	18.71	17.57
repl. 3	P-Akt	0.86	0.14	0.51	2.45	1.14	0.19	0.85	0	0.21	4.82	0.09	0
	Akt	0.63	0.37	0.4	0.17	0.38	0.35	1	0.73	0.68	1.87	2.03	1.77
	P-Erk	0.65	0.69	0.65	1.15	0.92	0.88	1.24	1.07	1.08	0.23	0.24	0.21
	Erk	5.59	2.76	2.97	1.83	2.05	1.93	1.46	1.18	1.43	3.01	2.6	2.52
	GAPDH	5.78	3.34	2.86	2.08	2.06	2.37	4.72	4.44	3.79	8.5	9.16	7.17
repl. 4	P-Akt	0.8	0.32	0	1.36	0	0	0.87	0.14	0.12	3.09	0.02	0.18
	Akt	0.27	0.71	0.73	0.42	0.38	0.36	0.84	0.73	0.54	1.05	1.06	1.37
	P-Erk	0.43	0.72	1	0.99	0.56	0.29	0.64	0.78	0.56	0.81	0.91	1
	Erk	2.44	1.95	3.34	2.33	1.14	0.93	1.19	1.81	1.06	1.8	2.55	2.89
	GAPDH	4.75	4.3	4.24	2.67	1.95	1.43	3.55	4.69	3.2	5.38	5.46	6.53
repl. 5	P-Akt	1.15	0	0	2.02	0.76	0.01	4.65	0.04	0.01	3.39	0	0
	Akt	0.97	1.1	0.63	0.52	1.96	0.69	1.9	0.43	1.22	1.77	1.66	1.8
	P-Erk	1.53	0.89	0.21	1.06	2.65	0.57	1.74	1.22	0.43	1.07	1.12	1.24
	Erk	7.71	5.94	3.38	2.94	7.03	2.16	2.14	1.27	0.87	3.89	3.83	4.05
	GAPDH	3.68	2.75	5.12	1.92	4.47	3.95	7.05	6.03	4.3	7.88	7.97	7.81
repl. 6	P-Akt	1.48	0	0	1.53	0.15	0.05	0.37	0	0.03	0.90	0.07	0.03
	Akt	1.87	0.97	2.62	0.77	0.61	0.66	0.82	0.62	0.78	0.69	0.56	0.50
	P-Erk	0.87	0.67	0.68	1.02	0.91	0.88	0.51	0.37	0.94	0.34	0.23	0.21
	Erk	5.27	3.11	2.80	1.67	1.47	1.67	1.82	1.25	1.53	2.04	1.64	1.40
	GAPDH	4.24	2.69	2.62	2.62	2.27	1.83	2.08	1.47	1.74	1.70	1.23	1.13

Table S2: Signal intensities from protein bands quantified in immunoblot analyses

		Regorafenib (µM)												Trametinib (µM)											
		HROG02			HROG05			HROG 15			HROG 24			HROG02			HROG05			HROG 15			HROG 24		
		0	10	30	0	10	30	0	10	30	0	10	30	0	1	3	0	1	3	0	1	3	0	1	3
repl. 1	P-Akt	2.65	3.45	3.11	6.23	5.19	4.7	2.99	0.18	0.05	4.24	2.62	2.8	1.09	6.78	9.56	6.43	7.5	6.39	4.08	5.78	6.5	5.89	9.67	6.02
	Akt	4.22	5.69	4.23	4.11	3.89	3.22	5.76	3.21	0.57	5.7	4.76	5.28	3.12	6.94	10.06	5.6	5.75	5.33	8.96	8.8	9.94	6.25	9.72	5.6
	P-Erk	2.73	3.21	1.32	2.58	1.95	1.17	0.89	0.39	0.46	0.97	0.62	0.11	5.46	0.436	0.796	2.26	0	0	2.42	0.544	0.214	2.03	0	0
	Erk	7.63	10.45	8.73	3.54	3.45	3.55	2.65	3.63	2.88	2.97	2.98	2.61	17.38	9.42	16.96	9.07	8.85	7.95	5.66	4.5	5.98	1.72	0.64	1.17
	GAPDH	5.79	6.3	5.96	4.38	4.33	4.36	16.2	16.2	14.4	3.19	3.3	2.41	16.72	7.92	16.14	7.26	6.58	5.62	33.2	26.6	26.8	5.64	8.8	7.29
repl. 2	P-Akt	1.66	2.18	4.08	4.21	3.39	6.57	1.57	0.56	0.46	4.98	2.66	4.94	6.48	6.44	3.74	10.73	12.68	15.97	1.07	1.83	4.06	6.37	13.45	11
	Akt	2.62	3.18	5.3	2.06	1.64	3.9	2.32	2.25	1.71	4.26	2.82	4.87	10.08	8.98	5.2	4.05	4.42	5.81	3.82	3.6	4.76	2.08	4.11	2.91
	P-Erk	3.42	3.66	1.58	2.07	0.94	0.84	0.48	0.43	0.11	1.48	2.04	0.5	2.32	0	0	18.93	20.9	24.6	0.84	0	0	4.24	0.5	0.1
	Erk	3.58	3.63	0.94	5.63	5.27	10	1.68	1.43	1.78	1.5	2.2	1.5	13.66	16.72	8.28	7.7	8.36	11.54	4.74	4.08	3.02	3.63	7.32	8.81
	GAPDH	8.13	4.72	6.63	4.38	4	3.85	11.9	10.6	8.7	7.32	4.9	7.56	15.48	17.8	12.48	7.41	8.65	9.65	21.8	20.6	17.64	7.02	7.23	7.7
repl. 3	P-Akt	2.45	3.13	3.26	1.85	5.27	5.43	1.09	0	0.53	1.92	3.5	2.1	5.78	6.18	7.88	5.76	8.49	4.53	3.78	2.88	3.8	0.72	1.52	0.71
	Akt	4.22	5.35	3.61	0.9	2.2	2.17	2.79	1.49	1.78	1.55	1.58	0.79	10.84	11.74	13.5	3.4	4.31	2.18	4.46	3.62	4.74	0.81	0.82	0.4
	P-Erk	3.06	3.69	1.4	4.49	2.96	0.94	0.48	0.62	0.28	5.35	6.27	5.61	3.9	0.35	0.20	6.32	0	0	1.16	0	0.03	3.2	0	0
	Erk	9.72	13.87	10.3	3.84	5.6	6.07	1.85	1.74	1.44	4.8	6.43	4.12	16.2	20	16.64	7.51	9.91	6.86	2.12	1.196	2.1	2.95	3.06	3.39
	GAPDH	7.57	5.98	4.57	4.21	6.13	5.48	12.9	8.44	8.74	1.65	2.32	1.84	15.66	19.12	26.8	8.22	8.47	5.96	15.9	12.58	12.38	2.56	2.77	3.4
repl. 4	P-Akt	1.72	2.86	3.41	3.93	4.91	2.84	1.8	0.43	0.31	3.6	3.01	1.59	3.94	1.65	1.78	2.1	2.4	2.04	4.2	5.48	5.66	3.68	2.91	4.18
	Akt	1.3	1.6	1.43	0.6	0.83	0.96	2.54	2.43	1.78	2.88	3.15	1.78	4.16	2	2.06	0.85	0.83	0.74	3.86	5.04	5.78	0.51	0.44	0.48
	P-Erk	5.76	5.19	3.54	1.9	0.83	1.89	0.91	0.47	0.04	1.37	2.02	0.48	2.86	0.76	0.33	1.63	0	0	1.23	0.06	0.31	0.32	0	0
	Erk	10.51	8.23	9.35	1.14	1.41	0.79	1.65	1.54	1.04	5.84	5.41	4.92	7.6	3.16	3.06	1.77	1.99	2.92	2.36	3.44	3.5	1.9	1.69	1.9
	GAPDH	4.15	4.05	6.11	8.24	6.68	6.17	11.2	9.02	8.94	4.1	5.03	2.6	11.06	7.58	6.18	3.01	1.35	2.73	16.4	20.8	20.8	5.39	4.89	5.47
repl. 5	P-Akt	2.04	2	2.44	2.94	3.14	3.6	2.64	0.78	0.50	12.28	13.6	12.92	0.85	3.56	3	4.61	3.62	5.49	6.2	6.78	5.24	1.49	2.54	3.21
	Akt	4.59	7.24	6.92	2.85	2.89	3.19	3.29	3.3	2.21	0.87	0.78	0.42	2.9	4	4.74	0.75	0.61	0.84	6.2	5.98	4.42	2.22	2.72	2.55
	P-Erk	13.5	14.28	5.53	2.33	3.4	3.01	0.66	0.29	0.03	3.39	0	0	4.16	0	0	1.22	0	0	0.99	0.08	0.58	3.21	3.87	4.08
	Erk	15.06	18.13	14.63	4	7.83	6.65	3.4	2.67	2.87	2.24	1.93	1.44	15.72	7.38	5.9	0.55	0.61	0.82	3.56	4.26	4.84	8.29	7.57	6.41
	GAPDH	13.86	10.52	13.15	1.61	10.11	8.23	12.5	10.6	10.4	7.4	8.38	7.24	13.32	9.12	11.18	4.93	7.59	5.84	15.9	19.04	19.42	8	7.62	6.77
repl. 6	P-Akt	8.21	6.67	7.86	7	9.23	8.37	1.8	1.89	0.97	7.51	4.91	5.29	2.56	8.72	3.62	5.15	12.52	11.99	8.18	8.9	3.22	3.63	6.34	8.66
	Akt	17.9	18.81	17.53	10.74	11.12	10.76	3.53	4.35	1.07	12.23	11.06	10.04	3.92	7.86	2.84	1.91	3.98	3.61	7.1	7.26	3.62	1.98	2.59	4.11
	P-Erk	6.57	6.21	2.61	2.26	1.93	0.36	0.86	0.82	0.04	2.92	1.12	0.97	4.56	0.31	0.97	2.22	0	0	1.19	0.50	0.58	1.3	0	0
	Erk	11.09	9.28	9.27	4.15	5.18	3.94	2.19	3.18	0.65	5.88	4.7	5.01	9.4	10.66	4.68	2.82	4.33	5.02	4.02	5.54	4.98	2.03	3.28	4.12
	GAPDH	21.96	19.12	18.68	6.76	9.32	5.98	9.15	5.83	4.32	8.19	6.8	6.23	7.72	9.92	4.14	3.34	6.45	5.92	16.74	17.52	19.64	4.23	4.3	7.09
repl. 7	P-Akt	3.5	4.16	5.79	6.28	6.59	7.12	2.69	0.76	1.07	5.49	4.34	4.18	3.02	6.18	7.58	13.86	18.18	15.38	4.5	5.06	7.12			
	Akt	3.19	4.07	3.52	1.97	1.82	1.67	2.75	2.45	1.72	2.3	1.96	1.82	5.18	7.9	10.38	5.41	6.31	5.46	4.3	6.84	5.18			
	P-Erk										1.46	0.75	0	2.96	0.15	0.07	2.79	0	0	1.10	0.04	0.10			
	Erk										2.74	4.38	2.86	7.76	13.54	11.88	5.42	6.45	6.06	3.54	3.18	4.26			
	GAPDH	7	3.87	2.96	2.36	2.21	1.4	8.56	7.91	7.07	4.55	5.39	2.32	10.6	12.8	15.7	9.67	8.13	7.79	9.6	13.38	19.58			
